# Reduced elastogenesis: a clue to the arteriosclerosis and emphysematous changes in Schimke immuno-osseous dysplasia?

**DOI:** 10.1186/1750-1172-7-70

**Published:** 2012-09-22

**Authors:** Marie Morimoto, Zhongxin Yu, Peter Stenzel, J Marietta Clewing, Behzad Najafian, Christy Mayfield, Glenda Hendson, Justin G Weinkauf, Andrew K Gormley, David M Parham, Umakumaran Ponniah, Jean-Luc André, Yumi Asakura, Mitra Basiratnia, Radovan Bogdanović, Arend Bokenkamp, Dominique Bonneau, Anna Buck, Joel Charrow, Pierre Cochat, Isabel Cordeiro, Georges Deschenes, M Semin Fenkçi, Pierre Frange, Stefan Fründ, Helen Fryssira, Encarna Guillen-Navarro, Kory Keller, Salman Kirmani, Christine Kobelka, Petra Lamfers, Elena Levtchenko, David B Lewis, Laura Massella, D Ross McLeod, David V Milford, François Nobili, Jorge M Saraiva, C Nur Semerci, Lawrence Shoemaker, Nataša Stajić, Anja Stein, Doris Taha, Dorothea Wand, Jonathan Zonana, Thomas Lücke, Cornelius F Boerkoel

**Affiliations:** 1 Provincial Medical Genetics Program, Department of Medical Genetics, Children's and Women's Health Centre of BC, 4500 Oak Street, Room C234, Vancouver, BC, V6H 3N1, Canada; 2 Rare Disease Foundation, Vancouver, British Columbia, Canada; 3 Department of Pathology, University of Oklahoma Health Sciences Center, Oklahoma City, Oklahoma, United States of America; 4 Department of Pathology, Oregon Health and Science University, Portland, Oregon, United States of America; 5 Department of Molecular and Human Genetics, Baylor College of Medicine, Houston, Texas, United States of America; 6 Department of Pathology, University of Washington, Seattle, Washington, United States of America; 7 Warren Clinic, Tulsa, Oklahoma, United States of America; 8 Department of Anatomic Pathology, University of British Columbia and Children’s and Women’s Health Centre of British Columbia, Vancouver, British Columbia, Canada; 9 Department of Medicine, University of Alberta, Edmonton, Alberta, Canada; 10 Department of Pediatrics, University of Oklahoma Health Sciences Center, Oklahoma City, Oklahoma, United States of America; 11 Néphrologie Pédiatrique, Hôpital d’Enfants, Centre Hospitalier Universitaire de Nancy, Vandoeuvre lés Nancy Cedex, France; 12 Department of Endocrinology & Metabolism, Kanagawa Children’s Medical Center, Yokohama, Japan; 13 Department of Pediatric Nephrology, Nephro-Urology Research Center, Shiraz University of Medical Sciences, Shiraz, Iran; 14 Institute of Mother and Child Healthcare of Serbia, Belgrade, Serbia; 15 Department of Pediatric Nephrology, VU University Medical Center, Amsterdam, The Netherlands; 16 Département de Génétique, Centre Hospitalier Universitaire d’Angers, Angers, France; 17 Medizinische Hochschule Hannover, Kinderklinik, Hannover, Germany; 18 Division of Genetics, Birth Defects and Metabolism, Children's Memorial Hospital, Chicago, Illinois, United States of America; 19 Centre de Référence des Maladies Rènales Rares, Hospices Civils de Lyon and Université de Lyon, Bron Cedex, France; 20 Serviço de Genética, Hospital Santa Maria, Centro Hospitalar Lisoboa Norte, Lisbon, Portugal; 21 Département de Pédiatrie, Hôpital Robert Debré, Paris, France; 22 Department of Internal Medicine, Division of Endocrinology and Metabolism, Cerrahi Hospital, Denizli, Turkey; 23 Pediatric Immunology & Hematology Unit, Necker Hospital, Paris, France; 24 Department of General Pediatrics, Pediatric Nephrology, University Children’s Hospital Münster, Münster, Germany; 25 Department of Medical Genetics, “Aghia Sophia” Children’s Hospital, Athens University Medical School, Athens, Greece; 26 Unidad de Genética Médica, Servicio de Pediatría, Hospital Universitario Virgen de la Arrixaca, Murcia, Spain; 27 Oregon Institute on Disability & Development, Child Development and Rehabilitation Center, Oregon Health & Science University, Portland, Oregon, United States of America; 28 Medical Genetics, Mayo Clinic, Rochester, Minnesota, United States of America; 29 Department of Genetics, Kaiser Permanente, San Francisco, California, United States of America; 30 Mercy Pediatrics and Adolescent Clinic, Clear Lake, Iowa, United States of America; 31 Department of Pediatric Nephrology, University Hospitals Leuven, Leuven, Belgium; 32 Department of Pediatrics, Immunology Program and Institute for Immunity, Transplantation, and Infection, Stanford University, Stanford, California, United States of America; 33 Divison of Nephrology, Bambino Gesù Children’s Hospital and Research Institute, Rome, Italy; 34 Department of Medical Genetics, Alberta Children’s Hospital, Calgary, Alberta, Canada; 35 Department of Nephrology, Birmingham Children’s Hospital, Birmingham, United Kingdom; 36 Service de Pédiatrie, Centre Hospitalier Régional Universitaire Hôpital Saint-Jacques, Besançon Cedex, France; 37 Consulta de Genética, Hospital Pediátrico de Coimbra, Coimbra, Portugal; 38 Department of Medical Genetics, Pamukkale University Hospital, Denizli, Turkey; 39 Division of Nephrology, Department of Pediatrics, Kosair Children’s Hospital, School of Medicine, University of Louisville, Louisville, Kentucky, United States of America; 40 Universitätsklinikum Essen, Kinderklinik, Essen, Germany; 41 Cape Breton Regional Hospital, Sydney, Nova Scotia, Canada; 42 Institut für Humangenetik, Martin-Luther-Universität Halle-Wittenberg, Halle, Germany; 43 Department of Neuropediatrics, Children’s Hospital, Ruhr-University Bochum, Bochum, Germany

**Keywords:** Schimke immuno-osseous dysplasia, *SMARCAL1*, Elastin, Vascular disease, Pulmonary emphysema

## Abstract

**Background:**

Arteriosclerosis and emphysema develop in individuals with Schimke immuno-osseous dysplasia (SIOD), a multisystem disorder caused by biallelic mutations in *SMARCAL1* (SWI/SNF-related, matrix-associated, actin-dependent regulator of chromatin, subfamily a-like 1). However, the mechanism by which the vascular and pulmonary disease arises in SIOD remains unknown.

**Methods:**

We reviewed the records of 65 patients with *SMARCAL1* mutations. Molecular and immunohistochemical analyses were conducted on autopsy tissue from 4 SIOD patients.

**Results:**

Thirty-two of 63 patients had signs of arteriosclerosis and 3 of 51 had signs of emphysema. The arteriosclerosis was characterized by intimal and medial hyperplasia, smooth muscle cell hyperplasia and fragmented and disorganized elastin fibers, and the pulmonary disease was characterized by panlobular enlargement of air spaces. Consistent with a cell autonomous disorder, SMARCAL1 was expressed in arterial and lung tissue, and both the aorta and lung of SIOD patients had reduced expression of elastin and alterations in the expression of regulators of elastin gene expression.

**Conclusions:**

This first comprehensive study of the vascular and pulmonary complications of SIOD shows that these commonly cause morbidity and mortality and might arise from impaired elastogenesis. Additionally, the effect of SMARCAL1 deficiency on elastin expression provides a model for understanding other features of SIOD.

## Background

Schimke immuno-osseous dysplasia (SIOD, OMIM 242900) is an autosomal recessive disorder associated with arteriosclerosis [1,2]. It is characterized by prominent skeletal dysplasia, renal failure, T-cell immunodeficiency, facial dysmorphism, and hyperpigmented macules [3-7]. Other features include osteoporosis, joint degeneration, hypothyroidism, abnormal dentition, bone marrow failure, thin hair, corneal opacities, atherosclerosis, cerebrovascular events (CVEs), and migraine-like headaches [2,6-10]. Severely affected patients usually die before 15 years of age from renal failure, infection, bone marrow failure, lung disease, or CVEs [7].

SIOD is caused by loss of function mutations in the gene encoding for the chromatin remodeling enzyme SWI/SNF-related, matrix-associated, actin-dependent regulator of chromatin, subfamily a-like 1 (SMARCAL1) [11]. SMARCAL1 functions as an annealing DNA helicase at single to double strand transitions in DNA [12] and as a DNA stress response protein [13,14]. It also interacts with replication protein A, participates in the resolution of stalled DNA replication forks, and modulates transcription [14-18]. Despite advances in our understanding of the SMARCAL1 enzyme, the mechanism by which SMARCAL1 deficiency leads to SIOD remains undefined.

As renal transplantation and dialysis have prolonged the longevity of SIOD patients, cerebral ischemia from arteriosclerosis has increasingly contributed to morbidity and mortality [7,19]. Although treatment with anticoagulant or hemorheological medications can transiently decrease the frequency and severity of CVEs and transient ischemic attacks (TIAs), the vascular disease ultimately progresses [7] and is not associated with detectable alterations in nitric oxide production or mitochondrial dysfunction [20,21]. Focal atherosclerotic plaques, generalized hyperplasia of the tunica media, and splitting and fraying of the internal elastic layer characterize the arterial pathology [1,2].

Potential contributors to the arteriosclerosis include hypertension, hyperlipidemia, renal disease, and immune dysfunction [3,7,22,23]. However, the arterial pathology observed by Clewing *et al.* is most similar to that reported for osteopontin deficiency or for impaired elastogenesis [1,24-26]. Osteopontin is a cytokine that is induced by the WNT signaling cascade [27], a cellular pathway that participates in the regulation of vascular smooth muscle cell proliferation [28]. Impaired elastogenesis arises either from mutations of *ELN* or from impaired function or expression of enzymes that process or bind elastin [29]. Mice heterozygous for *Eln* gene deletions show many features in common with SIOD patients, including systemic hypertension, pulmonary hypertension, aortic valve disease and frequent inguinal hernias [7,30,31]. Further highlighting the possibility of impaired elastogenesis, the postmortem lungs of two SIOD patients showed enlarged air spaces or emphysematous changes, a common feature in disorders of elastogenesis [7,30,31].

Given these observations, we hypothesized that osteopontin deficiency and/or impaired elastogenesis were the primary causes of the vascular and pulmonary disease associated with SIOD. To test these hypotheses and to determine the prevalence of vascular and pulmonary disease among SIOD patients, we reviewed the records of SIOD patients with identified *SMARCAL1* mutations, delineated the arterial and pulmonary pathology and profiled gene expression in postmortem artery and lung. We identify reduced elastin expression and synthesis as a possible basis of the arteriosclerosis and pulmonary emphysema of SIOD patients.

## Methods

### Patients

Patients referred to this study signed informed consent documents approved by the Institutional Review Board of Baylor College of Medicine (Houston, TX, USA) or the University of British Columbia (Vancouver, BC, Canada). Clinical data for 65 SIOD patients were obtained from questionnaires completed by the attending physician and from medical records and summaries provided by that physician. Autopsy tissues were obtained according to the protocol approved by the University of British Columbia. The *SMARCAL1* mutations of SIOD patients are listed in Table 1.

**Table 1:** **Summary of pulmonary and vascular findings in SIOD patients with *SMARCAL1 *** **mutations**

**Pedigree No.**	***SMARCAL1 *** **mutations**	**Sex**	**Pulmonary findings**		**Vascular findings**				**Age at death**	**Cause of death**
			**Age at onset**	**Lung dysfunction or pathology**	**Age at onset**	**CVA**	**TIA**	**Moya moya**		
SD4a	c.[1930C>T];[410delA]	F		NR		-	NR	NR	8	Renal failure
SD4b	c.[1930C>T];[410delA]	M		NR		-	NR	NR	8	Renal failure
SD8	c.[1190delT];[?]^A^	F		NR		-	NR	NR	5.7	Pneumonia
SD16	c.[1933C>T];[1643T>A]	M	34	Mild panlobular emphysema with dyspnea, pulmonary hypertension, restrictive lung disease		-	-	-		
SD18a	c.[1756C>T];[1756C>T]	M		NR		NR	-	NR	43	Cryptococcus meningitis
SD18c	c.[1756C>T];[1756C>T]	F		-		-	-	-		
SD22	c.[2459G>A];[2459G>A]	M		NR	8	-	+	-	14.6	CMV infection
SD23	c.[2542G>T];[2542G>T]	M		NR	4.1	+	+	-	10.3	Unknown
SD24	NT_005403.17: g.[67482574_67497178del] +[67482574_67497178del]	F		-	7.5	+	+	NR	9	CVE
SD25	c.[100C>T];[49C>T]	F		-	5	+	+	NR	10.1	CVE
SD26	c.[2542G>T];[1190delT]	M	NR	Pulmonary edema	5.3	+	+	-	8	Renal and bone marrow failure
SD27	c.[1940A>C];[1940A>C]	F		-		-	-	-	25.6	Infectious pulmonary disease
SD28	c.[1696A>T;1698G>C;1702delG]; [1696A>T;1698G>C;1702delG]	M	12	Chronic cough, dyspnea, pulmonary hypertension		NR	-	NR	12	Pulmonary hypertension
SD29	c.[1934delG];[862+1G>T]	M	3.7	Pulmonary edema, restrictive lung disease, pulmonary fibrosis	< 3	+	+	NR	4	Infectious pulmonary disease^B^
SD30	c.[1132G>T];[1132G>T]	F		NR	5.7	+	-	+	10	HSV pneumonitis
SD31	NT_005403.17: g.[67482574_67497178del] +[67482574_67497178del]	F		NR	11	+	+	+	14	Lymphoproliferative disease (secondary)
SD33a	c.[1146_1147delAA;1147+1_2delGT]; [1097-2A>G]	F		-		-	-	NR	2.8	Bone marrow failure
SD33b	c.[1146_1147delAA;1147+1_2delGT]; [1097-2A>G]	M		-	< 1	+	-	NR	3.7	CVE
SD35	c.[1736C>T];[2321C>A]	M	NR	Pulmonary fibrosis		-	-	-	8	Renal failure
SD38	c.[1096+1G>A];[1096+1G>A]	M	2	Asthma	NR	NR	+	-	10.8	Complications of blood stem cell transplant
SD39	c.[2114C>T];[1402G>C]	M		NR	11	+	+	NR	15	CVE
SD44	c.[2321C>A];[1191delG]	M		-	9	+	+	NR	11.9	Digestive bleeding
SD47	c.[2459G>A];[?]^A^	M		-	7	+	+	-		
SD48	c.[1939A>C];[1939A>C]	F	6.8	Pulmonary hypertension	4	+	+	+	6.8	EBV pneumonia
SD49	c.[2321C>A];[1920_1921insG]	M	4.8	Pulmonary edema		-	-	-	4.8	Unknown
SD50	c.[2542G>T];[2542G>T]	F	3	Restrictive lung disease	4.5	+	+	NR	8	Peritonitis and sepsis post transverse colon perforation
SD51	c.[2542G>T];[2459G>A]	F		-		-	-	-		
SD53	c.[2291G>A];[2543G>T]	M		NR		-	-	NR		
SD57	c.[955C>T];[955C>T]	F	< 10	Asthma, wheezing, basilar atelectasia	8	+	+	-	28	Pancreatitis
SD60	c.[2542G>T];[2542G>T]	M	NR	Pulmonary edema, pulmonary hypertension, emphysematous changes upon autopsy	8	+	+	NR	13.7	CVE
SD61	c.[1146_1147delAA;1147+1_2delGT]; [1146_1147delAA;1147 + 1_2delGT]	M		-		-	-	NR	5	Lymphoproliferative disease (primary)
SD65a	c.[2542G>T];[836T>C]	M		-		-	-	-		
SD65b	c.[2542G>T];[836T>C]	M	23	Diffusion and perfusion lung disorder	14	+	+	-		
SD66	c.[1933C>T];[1933C>T]	M	NR	Pulmonary edema	7	+	+	+	13	Congestive heart failure
SD68	c.[1940A>C];[2462T>G]	F		-	6	+	+	-	7.1	CVE
SD70	c.[340_341insAGTCCAC];[836T>C]	F		-	6	+	+	-	18	Recurrent ileus pathology
SD71	c[1000C>T];[836T>C]	M		-	6	+	+	-	9	Unknown
SD74	c.[1736C>T];[?]^A^	M		-		-	-	-		
SD78	c.[2264T>G];[1439C>T]	F		-		NR	-	NR	10	Pneumonia
SD79	c.[2459G>A];[?]^A^	F		-		-	-	-	10	Complications of BMT
SD84	c.[2104T>G];[1248_1249insC]	M	NR	Pulmonary hypertension, emphysematous changes upon autopsy	10	-	+	+	23	Pulmonary hypertension with heart failure
SD86	c.[2263_2282delATCGATGGCTCCACCTCATC];[1129G>C]	F		-		-	-	-	5.7	Complications following BMT
SD96	c.[1427G>A];[1427G>A]	M	6	Recurrent lung infections		-	-	-	6	Infection^C^
SD99	c.[1402G>C];[1402G>C]	F	5.5	Pulmonary edema		-	-	NR	5.5	Pulmonary edema and left heart failure
SD101	c.[2542G>T];[2542G>T]	M		NR	3	-	+	-		
SD102	c.[2542G>T];[2542G>T]	M		NR		NR	NR	NR	8	Renal failure
SD106	c.[1682G>A];[1682G>A]	M	4	Chronic coughing and wheezing	5.5	-	+	NR	8	Non-Hodgkin Lymphoma
SD107	c.[2542G>T];[2542G>T]	F	3	Restrictive lung disease		NR	-	NR	6	Thrombosis
SD108a	c.[1798C>T];[1798C>T]	M		-		-	-	-		
SD108b	c.[1798C>T];[1798C>T]	M		-		-	-	NR		
SD111	c.[1129G>C];[1592T>C]	M	13	Pulmonary hypertension, chronic cough, restrictive lung disease	15	+	+	-	17.5	Respiratory failure
SD112a	c.[1934G>A];[2542G>T]	F		-		-	-	-		
SD112b	c.[1934G>A];[2542G>T]	F		-		-	-	-		
SD114	c.[1898T>C];[1898T>C]	M		-	4	+	-	+	9.5	Unknown
SD115	c.[1437_1438insG];[1437_1438insG]	F	< 0.6	Mild bronchiectasis		-	-	-	1	Pneumonia with respiratory failure
SD119	c.[2449C>T];[2542G>T]	F		NR		NR	-	NR		
SD120	c.[2291G>A];[2542G>T]	M	NR	Restrictive lung disease, emphysematous changes	3	-	+	-	5.5	Respiratory failure
SD121	c.[1382G>A];[2542G>T]	F		-	3.3	+	-	-	4.8	CVE
SD123	c.[49C>T];[49C>T]	F		-	4	-	+	-		
SD124	c.[1920_1921insG];[1920_1921insG]	M		-		-	NR	-		
SD127	c.[1736C>T];[1736C>T]	F	9	Reactive airway disease	7	+	+	+		
SD131	c.[1026C>A];[2264T>G]	M		-	NR	+	-	-	4.6	Cerebral hemorrhage
SD133a	c.[863-2A>G;2343_2347_delGCTGT]; [=;2343_2347_delGCTGT]	F		-		-	-	-	3	Pulmonary embolism (secondary)
SD133b	c.[863-2A>G;2343_2347_delGCTGT]; [=;2343_2347_delGCTGT]	F								Terminated pregnancy
SD138	c.[2542G>T];[2542G>T]	M		-	NA		NA	NA	NA	

Abbreviation: +, feature present; -, feature not present; BMT, bone marrow transplant; CVE, cerebrovascular event; CMV, cytomegalovirus; EBV, Epstein-Barr virus; F, female; HSV, herpes simplex virus; M, male; NA, not applicable; NR, not reported; TIA, transient ischemic attack.

^A^[?] represents alleles with noncoding *SMARCAL1* mutations as described by Clewing *et al*. [60].

^B^No bacteriologic or viral proof.

^C^Infection of peritoneal dialysis fluid.

### Case reports

#### Patient SD120

The propositus was a 5.4-year old boy with Schimke immuno-osseous dysplasia (Additional file 1). He was born at 35 weeks gestation to healthy non-consanguineous parents by Cesarean section for intrauterine growth retardation and oligohydramnios. At 1 year of age, he underwent surgery to repair an inguinal hernia. At 3.2 years of age, he had surgery for bilateral hip dysplasia. Beginning in his third year, he developed recurrent migraine-like headaches with aura, vomiting and hemiplegia. By 4 years of age, he manifested recurrent TIAs, although he had normal brain magnetic resonance imaging and angiography as well as normal electroencephalogram studies. At 4.5 years of age, he developed nephrotic syndrome that progressed to end-stage renal disease requiring dialysis. At 5.4 years, he was admitted to hospital for fever, anemia, hypoxia, and respiratory distress. His respiratory status rapidly worsened despite antibiotics and mechanical ventilation. He died from respiratory failure without identification of its cause. Postmortem studies showed alveolar damage, alveolitis, bronchitis, and dilated air spaces as well as arteriosclerosis, atherosclerosis, and cardiac left ventricular hypertrophy.

#### Patients SD60 and SD84

SD60 was a 13.7-year old boy and SD84 was a 23-year old man. Both have been described previously [1,2].

#### Patient SD16

**Figure 1:**
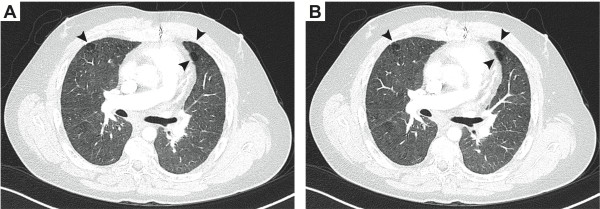
**Emphysematous lung changes in an SIOD patient.** (**A**, **B**) Consecutive axial computerized tomography images of the chest of patient SD16 at age 34 years. The images were captured 1 mm apart. Note the lung blebs (arrows).

The propositus is a 36-year old man with mild SIOD (Additional file 1); he was described by Gilchrist *et al.* at 16 years of age [32]. Since then, he has had bilateral hip and aortic valve replacement and respiratory insufficiency requiring oxygen supplementation. He has no history of smoking or exposure to cigarette smoke. His spirometry and diffusion studies show signs of both restrictive and obstructive pulmonary disease (Additional file 2). The former is consistent with his skeletal dysplasia and the latter is explained by the mild panlobular emphysema identified by computed tomography (Figure 1A, B). The severity of his respiratory distress has been disproportionate to his lung pathology, and is explained by Type I pulmonary arterial hypertension detected on right heart catheterization.

### RNA isolation and reverse transcription

For cultured cells, RNA was extracted from 1 X 10^7^ cells using the RNeasy Mini Kit (Qiagen, Mississauga, ON, Canada). For tissues, RNA was extracted from flash frozen tissue pulverized with a Bessman tissue pulverizer and lysed with TRIzol reagent (Invitrogen, Burlington, ON, Canada) according to the manufacturer’s specifications. Subsequently, the RNeasy Mini Kit (Qiagen, Mississauga, ON, Canada) was used to purify the RNA. Residual genomic DNA was removed by DNase I digestion.

Control aorta RNA pooled from 4 unaffected individuals ranging in age from 27–45 years was purchased from Clontech (636546, Lot no. 9052725A, Mountain View, CA, USA). Control lung RNA pooled from 3 unaffected individuals ranging in age from 32–61 years was purchased from Clontech (636643, Lot no. 8101369A, Mountain View, CA, USA).

RNA from formalin-fixed paraffin-embedded umbilical cord was isolated using the Ambion RecoverAll Total Nucleic Acid Isolation Kit (AM1975, Life Technologies, Burlington, ON, Canada) according to the manufacturer’s specifications.

Reverse transcription was performed with the qScript™ cDNA Synthesis Kit (Quanta Biosciences, Gaithersburg, MD, USA) or the RT^2^ First Strand Kit (SABiosciences, Mississauga, ON, Canada) using 500 ng of RNA per reaction according to the manufacturer’s specifications.

### PCR

Following reverse transcription, 1.5 μl of cDNA served as template for each reaction and was amplified with the HotStarTaq Master Mix Kit (Qiagen, Toronto, ON, Canada). The following conditions were used for amplification: 1 cycle of 95°C for 15 min, followed by 30 cycles of 94°C for 30 s, 55°C for 30 s, 72°C for 1 min, and a final extension at 72°C for 10 min. PCR was performed using the primers listed in Additional file 3.

### Gene expression array

The Atherosclerosis (PAHS-038) RT^2^ Profiler™ PCR Array from SABiosciences (Mississauga, ON, Canada) was used to assess differences in gene expression between control and SIOD aortic mRNA according to the manufacturer’s instructions.

### Quantitative PCR

SsoFast EvaGreen Supermix (Bio-rad Laboratories, Mississauga, ON, Canada) or RT^2^ Real-Time™ SYBR Green/Rox PCR master mix (SABiosciences, Mississauga, ON, Canada) was used with the ABI 7500 Fast Real-Time PCR System for quantitative PCR. The primer sequences are listed in Additional file 3.

### *ELN* mutation analysis

Genomic DNA was extracted from the aorta of SD120 using the DNeasy Tissue Kit (Qiagen, Toronto, ON, Canada) according to the manufacturer’s specifications. The 34 exons of *ELN* were amplified with the HotStarTaq Plus Master Mix Kit (Qiagen, Toronto, ON, Canada). The following conditions were used for amplification: 1 cycle of 95°C for 5 min, followed by 35 cycles of 94°C for 30 s, 55°C or 60°C for 30 s, 72°C for 45 s, and a final extension at 72°C for 10 min. PCR was performed using the primers listed in Additional file 3. Unincorporated primers and nucleotides were removed using ExoSAP-IT reagent (USB, Cleveland, OH, USA).

Sanger capillary sequencing was used to sequence the PCR products (Macrogen, Seoul, Korea), and the sequences were aligned and analyzed using Sequencher v.4.10.1 (Gene Codes, Ann Arbor, MI, USA). Mutation interpretation analysis was conducted using Alamut 2.0 (Interactive Biosoftware, San Diego, CA, USA).

### Cell culture

Aortic smooth muscle cells (AoSMCs, CC-2571, Lonza, Walkersville, MD, USA) were grown in smooth muscle basal medium (SmBM) supplemented with 5% fetal bovine serum (FBS), epidermal growth factor (EGF), basic fibroblast growth factor (FGF-B), insulin, gentamicin, and amphotericin B (SmGM-2 BulletKit, CC-3182, Lonza, Walkersville, MD, USA).

Human iliac artery endothelial cells (HIAECs, CC-2545, Lonza, Walkersville, MD, USA) were grown in endothelial basal medium (EBM-2) supplemented with 5% FBS, EGF, FGF-B, vascular endothelial growth factor (VEGF), R^3^ insulin-like growth factor 1 (R^3^-IGF-1), hydrocortisone, ascorbic acid, gentamicin, and amphotericin B (EGM-2-MV BulletKit, CC-3202, Lonza, Walkersville, MD, USA).

Aortic adventitial fibroblasts (AoAFs, CC-7014, Lonza, Walkersville, MD, USA) were grown in stromal cell basal medium (SCBM) supplemented with 5% FBS, FGF-B, insulin, gentamicin, and amphotericin B (SCGM BulletKit, CC-3205, Lonza, Walkersville, MD, USA).

Normal human lung fibroblasts (NHLFs, CC-2512, Lonza, Walkersville, MD, USA) were grown in fibroblast basal medium (FBM) supplemented with 2% FBS, FGF-B, insulin, gentamicin, and amphotericin B (FGM-2 BulletKit, CC-3132, Lonza, Walkersville, MD, USA).

### Immunofluorescence

Immunostaining of cultured cells was performed as previously described [33]. 5 x 10^5^ cells were grown overnight on a coverslip in a 6-well plate. With the exception of the aortic smooth muscle cells (AoSMCs), all cells were fixed with 4% paraformaldehyde (PFA) for 15 min at room temperature and permeabilized with 0.5% Triton X-100 for 15 min at room temperature. AoSMCs were fixed with 4% PFA and 0.15% picric acid for 20 min at room temperature, and permeabilized with 0.1% Triton X-100, 1% bovine serum albumin (BSA), and 10% normal horse serum in 1X phosphate buffered saline (PBS). All cells were blocked overnight with Blocker Casein in PBS (Pierce, Rockford, IL, USA) containing 10% normal horse serum at 4°C. The cells were then incubated with anti-SMARCAL1 (1:200) [33], anti-α-smooth muscle actin (1:20, 1A4, Dako, Mississauga, ON, Canada), anti-VE-cadherin (1:100, 33E1, Leica, Richmond Hill, ON, Canada), anti-prolyl 4-hydroxylase (1:50, 5B5, Abcam, Cambridge, MA, USA), or anti-α-tubulin (1:400, DM 1A, Sigma-Aldrich, Oakville, ON, Canada) diluted in blocking buffer at 4°C for 24 h. Cells then were gently washed 4 times with PBS and incubated with Alexa Fluor-conjugated secondary antibodies Alexa 488 and Alexa 555 (1:1000, Molecular Probes, Burlington, ON, Canada) for 1 h at room temperature. Cells next were washed 4 times with PBS and mounted in Vectashield containing 4’,6-diamidino-2-phenylindole (DAPI, Vector Laboratories, Burlington, ON, Canada). Images were acquired using a 100×/1.30 oil Plan-NEOFLUAR objective lens, a Zeiss Axiovert 200 inverted microscope, a Zeiss AxiocamMR camera, and the Zeiss Axiovision imaging system.

### Immunoblot analysis

Immunoblot analysis on cell lysates was performed as previously described [33]. Cell lysates were fractionated by 12% sodium dodecyl sulfate polyacrylamide gel electrophoresis (SDS-PAGE) and transferred to a polyvinylidene fluoride (PVDF) membrane. The membrane was blocked overnight at 4°C, using gentle agitation, in PBS containing 0.2% I-Block (Applied Biosystems, Foster City, CA, USA) and 0.1% Tween 20 overnight. Anti-SMARCAL1 (1:2000) [33] and anti-glyceraldehyde 3-phosphate dehydrogenase (GAPDH, 1:2000, 6C5, Advanced ImmunoChemical Inc., Long Beach, CA, USA) were used as primary antibodies. Alkaline phosphatase-conjugated secondary antibodies (1:10000, Bio-rad Laboratories, Mississauga, ON, Canada) were used to detect the primary antibodies. The bound antibody was detected by chemiluminescence using CDP-Star (Applied Biosystems, Streetsville, ON, Canada) according to the manufacturer’s specifications. GAPDH was detected as a loading control.

Immunoblot analysis on human tissue was performed as previously described [33]. Anti-elastin binding protein (EBP, 1:200, a kind gift from Dr. Amelia Morrone, University of Florence, Florence, Italy) [34,35] and anti-GAPDH were used as primary antibodies. EBP expression in the aortas of two SIOD patients was compared to that of a control aorta protein medley pooled from 49 unaffected individuals ranging in age from 15–65 years and purchased from Clontech (635310, Lot no. 5110079, Mountain View, CA, USA). EBP expression was normalized to expression of GAPDH for each sample. Densitometry of three independent replicates was conducted using the Kodak 1D Image Analysis Software version 3.6.

### Tissue immunohistochemistry and staining

Formalin-fixed, paraffin-embedded sections were cut at 5 microns. Following deparaffinization and rehydration, heat induced epitope retrieval was conducted with sodium citrate buffer (10 mM sodium citrate, 0.05% Tween 20, pH 6) or tris-ethylene diamine tetraacetic acid (EDTA) buffer (10 mM Tris base, 1 mM EDTA, 0.05% Tween 20, pH 9). For immunohistochemical detection of elastin, proteolytic induced epitope retrieval was conducted with 0.4% pepsin at 37°C for 15 min. Endogenous peroxidases were inactivated by preincubating the sections with 0.3% H_2_O_2_ in methanol. Non-specific protein binding was blocked by preincubation at 4°C with blocking buffer (10% horse serum in TBS-T (10 mM Tris–HCl, 150 mM NaCl, 0.01% Tween 20, pH 7.4)) for 24 h. Sections were then incubated with anti-SMARCAL1 (1:200) [33], anti-CD3 (1:50, MRQ-39, Cell Marque, Rocklin, CA, USA), anti-CD20 (1:50, L26, Cell Marque, Rocklin, CA, USA), anti-CD68 (1:250, KP1, Dako, Mississauga, ON, Canada), anti-α-smooth muscle actin (1:500, 1A4, Dako, Mississauga, ON, Canada), or anti-elastin (1:50, BA-4, Abcam, Cambridge, MA, USA) diluted in blocking buffer at 4°C for 24 h. Sections were then washed 5 times with TBS-T and incubated with horseradish peroxidase (HRP)-conjugated secondary antibodies (EnVision+ System, Dako, Mississauga, ON, Canada) for 30 min at room temperature. Sections were then washed 3 times with TBS-T and 3,3’-diaminobenzidine (DAB, EnVision+ System, Dako, Mississauga, ON, Canada) was subsequently used as an HRP substrate. Sections were counterstained in Mayer’s Hematoxylin (Sigma, Oakville, ON, Canada).

Histochemical stains on tissue sections included a modified Verhoeff van Geison elastic stain (HT25A, Sigma, Oakville, ON, Canada) for elastic fibers and a periodic acid-Schiff stain (395B, Sigma, Oakville, ON, Canada) for neutral glycosaminoglycans. Images were acquired using a 5x/0.15 Plan-NEOFLUAR, 10x/0.45 Plan-APOCHROMAT, 20x/0.75 Plan-APOCHROMAT, 63x/1.4 oil Plan-APOCHROMAT, or 100x/1.30 oil Plan-NEOFLUAR objective lens on a Zeiss Axiovert 200 inverted microscope, a Zeiss AxiocamHR camera, and the Zeiss Axiovision imaging system.

### Fastin elastin assay

The elastin content of arterial tissue was quantified using the Fastin Elastin Assay Kit (F2000, Bicolor Life Science Assays, United Kingdom). Tissue samples were flash frozen and pulverized with a Bessman tissue pulverizer, weighed and digested with 0.25 M oxalic acid at 95°C for six 1 h time periods. Elastin concentration in pooled supernatants was calculated from the elastin standard curve and the total elastin per wet weight of each sample was determined according to the manufacturer’s specifications.

### Arterial thickness analysis

Analysis of the aortic intimal and medial thickness was carried out using the Zeiss Axiovision imaging system and software to measure the width of the tunica intima and the tunica media. Four random images of each sample were taken and 5 random measures were taken for both the tunica intima and tunica media for each image. Measures of the tunica intima were taken from the luminal edge of the endothelium to the internal elastic lamina perpendicular to the internal elastic lamina; measures of the tunica media were taken from the internal elastic lamina to the boundary between the tunica media and the tunica adventitia perpendicular to the internal elastic lamina. Ratios were calculated comparing the widths of the tunica intima and tunica media of SIOD patient aortas to that of age-matched control aortas.

### Echocardiogram measurements

A transthoracic echocardiogram was obtained by a Philips iE33 echocardiography machine using an S8 phased array ultrasound transducer probe with the patient in the supine and left lateral position. M-mode, 2D, color Doppler, pulse wave Doppler and continuous wave Doppler were obtained. Standard views including long axis view, short axis view, four chambers, subcostal and suprasternal notch views were obtained.

The following measurements of the aorta in systole were obtained in accordance to the American Society of Echocardiogram guidelines in real time and confirmed postmortem [36]: the aortic valve, the aortic root at the sinus of Valsalva, the sinotubular junction and the ascending aorta. Body surface area and Z scores were calculated offline using the Haycock and Halifax formula, respectively [37,38].

### Pulmonary function testing

Patient SD16 performed complete pulmonary function tests that met the American Thoracic Society (ATS) criteria for acceptability [39]. This included spirometery, lung volumes measured via plethsysmography, and diffusion capacity measured via nitrogen washout.

### Statistical analysis

Quantitative data are presented as the mean ± 1 standard deviation calculated from a minimum of 3 independent replicates. Data were analyzed by the paired 2-tailed Student’s *t*-test or the one-way analysis of variance (ANOVA) followed by the Tukey post hoc test where appropriate. A p-value of less than 0.05 was considered statistically significant.

## Results

### Pulmonary and vascular disease is common in SIOD

Among SIOD patients with *SMARCAL1* mutations, 22 of 51 (43.1%) patients had lung disease (Table 1). Obstructive lung disease was present in 7 (13.7%) patients, including 3 (5.9%) with asthma or reactive airway disease, 1 (2.0%) with bronchiectasis, and 3 (5.9%) with emphysematous changes (Table 1).

Regarding the vascular disease, 32 of 63 (50.8%) patients had clinical symptoms of cerebral ischemia and 6 of 51 (11.8%) patients had documented pulmonary hypertension. Twenty-five of 58 (43.1%) patients had CVEs and 27 of 59 (45.8%) patients had TIAs (Table 1). For 7 patients, the onset of cerebral ischemia preceded the development of renal disease or hypertension, and among patients who received a renal transplant, cerebral ischemia worsened despite renal transplantation (data not shown).

Of the 65 patients with *SMARCAL1* mutations, 47 have died. Six (12.8%) died from pulmonary complications, and 7 (14.9%) died from vascular disease (Table 1).

### Histopathology of the SIOD aorta shows fragmented elastin fibers and hyperplasia of the tunica intima and media

**Figure 2:**
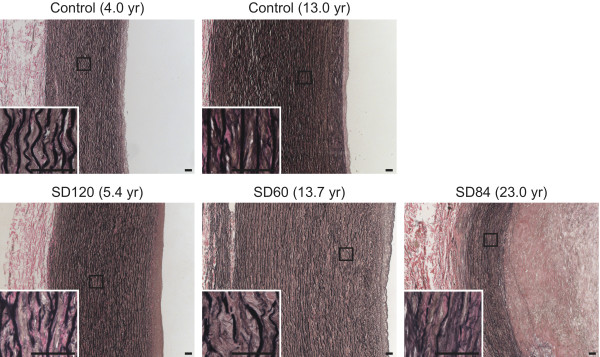
**Photomicrographs of Verhoeff van Gieson stained aortas from SIOD patients and age-matched controls.** Compared to age-matched controls, note the decreased elastin fiber staining, the fragmentation and splitting of the elastin fibers, the marked hyperplasia of the tunica intima and the tunica media in the aorta from three individuals with SIOD. Arteries are oriented with the tunica adventitia on the left and the tunica intima on the right; the age of death is in parentheses. Scale bars: 50 μm.

To define better the histopathology of SIOD blood vessels, we analyzed postmortem arterial tissue from three individuals with SIOD (SD60, SD84 and SD120). Verhoeff van Gieson staining showed fragmented elastin fibers compared to age-matched controls (Figure 2 and Additional file 4). The aorta, common iliac, and pulmonary arteries of SD60, SD84 and SD120 had marked intimal and medial hyperplasia accompanied by an increased number of elastic lamellae compared to age-matched controls (Figure 2 and Additional file 4). The aortic tunica intima of SD60 and SD120 were 2.6-fold (p-value = 3.5 × 10^-13^) and 1.4-fold (p-value = 2.1 × 10^-3^) thicker than age-matched controls, respectively; the tunica media of SD60 and SD120 were 1.3-fold (p-values = 5.6 × 10^-21^ and 7.2 × 10^-15^, respectively) thicker than age-matched controls. By echocardiogram, SD120 had increased diameter of the sinotubular junction compared to normal using the Halifax formula, although measures at other levels of the aortic root were within the normal range (Additional file 5). Immunostaining for α-smooth muscle actin showed an increased number of positive cells suggesting smooth muscle cell hyperplasia in the aortic intima of SD60 and SD120 (Additional file 6).

### Inflammation is not increased in the SIOD aorta

Since SIOD patients have an immune disorder and the inflammation of atherosclerosis causes smooth muscle cell hyperplasia [40,41], we also looked for evidence of arterial inflammation. Using CD68 as a macrophage marker, CD3 as a T-cell marker, and CD20 as a B-cell marker, immunostaining did not detect an inflammatory infiltrate within the arterial walls (Additional file 7) except for the CD68^+^ macrophages within the atherosclerotic lesions of SD84 (Additional file 7).

### Histopathology of the SIOD umbilical cord shows a fragmented internal elastic lamina

**Figure 3:**
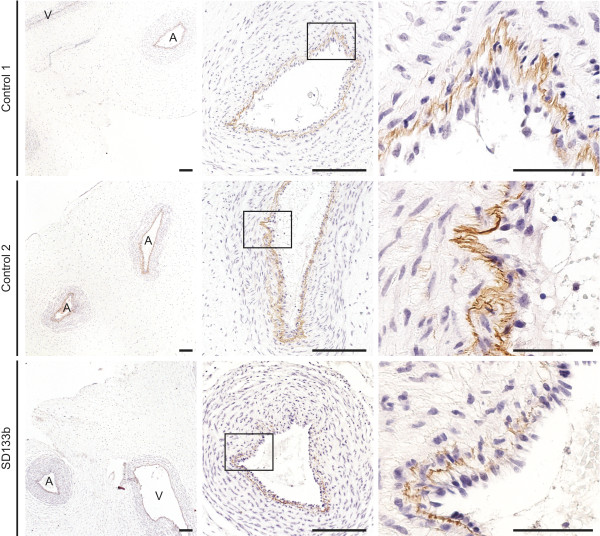
**Elastin expression analysis of the umbilical cord from SIOD and unaffected fetuses at 15-weeks gestation.** Note that the immunohistochemical analysis shows marked discontinuity and reduced expression of elastin in the internal elastic lamina in SD133b compared to that of 2 age-matched controls. This difference in expression of elastin precedes the development of hypertension, hypercholesterolemia, and renal disease. Abbreviations: A, artery; V, vein. Scale bars: 50 μm.

As longstanding hypertension, renal failure and hyperlipidemia of individuals SD60, SD84 and SD120 could be a cause of the arterial disease and are not present in individuals with SIOD at birth, we tested this hypothesis by studying the umbilical artery of a 15-week gestation fetus (SD133b) with biallelic *SMARCAL1* mutations (Table 1). Compared to age-matched controls, the fetal umbilical arteries of SD133b had interrupted circumferential expression of tropoelastin and elastin suggesting an intrinsic problem with elastogenesis (Figure 3). Moreover, analysis of *ELN* mRNA expression in the umbilical cord of SD133b and two age-matched controls showed that the umbilical cord of both controls had 1.75- to 2.95-fold higher *ELN* mRNA expression compared to that of SD133b (Additional file 8).

### SMARCAL1 is expressed in the vascular smooth muscle, endothelial, and adventitial fibroblast cells of the arterial wall

**Figure 4:**
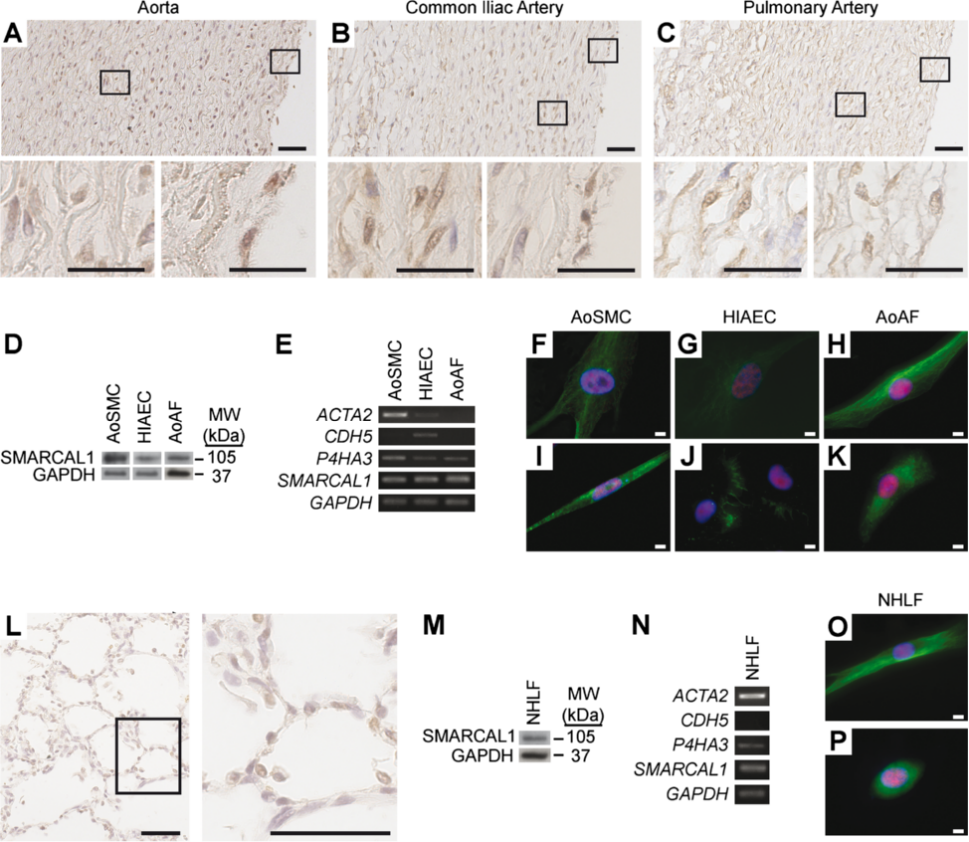
***SMARCAL1 *mRNA and protein are expressed in arterial and pulmonary tissue.** (**A**-**C**) Photomicrographs of immunohistochemical detection of SMARCAL1 in the aorta, common iliac and pulmonary arteries. (**D**) Photograph of an immunoblot showing expression of SMARCAL1 in aortic smooth muscle cells (AoSMCs), human iliac artery endothelial cells (HIAECs) and aortic adventitial fibroblasts (AoAFs). (**E**) Photograph of an agarose gel of RT-PCR products showing expression of *SMARCAL1* mRNA and cell-specific markers in AoSMCs, HIAECs, and AoAFs. Note that smooth muscle actin (*ACTA2*) is a marker of myofibroblasts and smooth muscle cells; VE-cadherin (*CDH5*) is a marker of endothelial cells; and prolyl 4-hydroxylase (*P4HA3*) is expressed in fibroblasts as well as multiple other cell types [59]. (**F**-**H**) Photomicrographs showing immunofluorescent localization of SMARCAL1 (red) and α-tubulin (green) in cultured AoSMCs, HIAECs, and AoAFs. (**I**-**K**) Photomicrographs showing immunofluorescent localization of SMARCAL1 (red) and the cell-specific markers smooth muscle actin (I), VE-cadherin (J), and prolyl 4-hydroxylase (K) in AoSMCs, HIAECs, and AoAFs (green), respectively. (**L**) Photomicrograph of immunohistochemical detection of SMARCAL1 in the lung. (**M**) Photograph of an immunoblot showing SMARCAL1 expression in normal human lung fibroblasts (NHLFs). (**N**) Photograph of an agarose gel of RT-PCR products showing expression of *SMARCAL1* mRNA and cell-specific markers in NHLFs. *GAPDH* was used as a control. (**O**) Photomicrographs showing immunofluorescent localization of SMARCAL1 (red) and α-tubulin (green) in cultured NHLFs. (**P**) Photomicrographs showing immunofluorescent localization of SMARCAL1 (red) and prolyl 4-hydroxylase (green) and in NHLFs. Scale bars: (A-C, L) 50 μm, 25 μm for inset; (F-K, O, P) 10 μm.

The above findings suggest a local or cell autonomous basis for the arteriosclerosis in SIOD, and consistent with this mechanism, arteriosclerosis does not recur in the transplanted kidneys of SIOD patients [1,2]. As a first requirement for a cell autonomous mechanism, SMARCAL1 must be expressed within the affected tissues, and indeed, SMARCAL1 was expressed in the adventitial fibroblasts, smooth muscle cells, and endothelium of the normal human aorta, common iliac and pulmonary arteries (Figure 4A-C). It was also expressed in the nuclei of cultured aortic smooth muscle cells (AoSMCs), iliac artery endothelial cells (HIAECs), and aortic adventitial fibroblasts (AoAFs) (Figure 4D-K and Additional file 9). Given these findings, we hypothesized that the cell autonomous mechanisms of osteopontin deficiency or impaired elastogenesis could give rise to arteriosclerosis.

### Osteopontin expression is not decreased in the SIOD aorta

**Figure 5:**
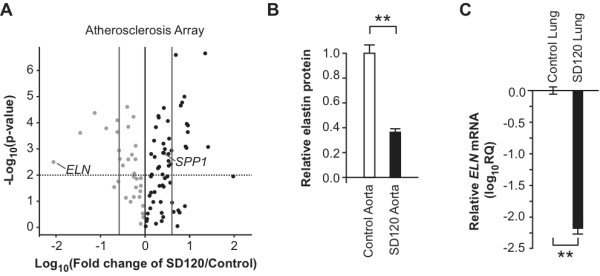
**Elastin expression is significantly decreased in the aorta and lung of an SIOD patient.** (**A**) Volcano plot comparing expression of atherosclerosis-related genes in the aorta of SD120 to control aorta. Note the markedly reduced expression of elastin (*ELN*). Solid grey lines: 4-fold change; solid black line: no change; dotted line: p = 0.01. Grey dots depict genes with decreased expression and black dots depict those with increased expression. (**B**) Relative elastin protein in the aortic wall of SD120 compared to control. Total elastin protein was measured with the Fastin Elastin Assay. Error bars represent one standard deviation. (**C**) Plot showing the level of *ELN* mRNA in SD120 and control lung tissue measured by qRT-PCR. The mRNA levels were standardized to *GAPDH* mRNA levels and plotted relative to the control. Note the markedly decreased *ELN* expression. Error bars represent one standard deviation. ** = p < 0.01.

Osteopontin is a direct target of the WNT pathway and deficient WNT signaling can lead to osteopontin deficiency [27]. Therefore, since the histopathology of aortas from *Opn*^*−/−*^*;Ldlr*^*−/−*^ mice resembles that of SIOD patients [26], we measured levels of *SPP1* mRNA, which encodes osteopontin. Contrary to our hypothesis, *SPP1* mRNA levels were increased by 2.6-fold in the aorta of SD120 compared to controls (Figure 5A and Additional file 10).

### Elastin binding protein is not decreased in the SIOD aorta

Based on the preceding, we hypothesized that the arteriosclerosis primarily arose from a defect of elastogenesis and was accentuated by hypertension, hyperlipidemia, and renal failure. One mechanism for impaired elastin fiber assembly is a reduction in the protective chaperone elastin binding protein (EBP) [42,43]. Elevated levels of glycosaminoglycans, which are found in the mucopolysaccharidoses like Morquio syndrome, Costello syndrome and Hurler’s disease, induce premature shedding of EBP and lead to impaired elastin fiber assembly [44-46]. Although later studies have not confirmed mucopolysacchariduria as a consistent feature of SIOD [6], we tested EBP levels since chondroitin-6-sulphaturia was initially described as a feature of SIOD by Schimke *et al.*[5]. Immunoblotting showed that EBP levels in the aortic lysate from SD60 and SD120 were comparable to those of controls (Additional file 11), and PAS staining did not show evidence of the increased deposition of neutral glycosaminoglycans that is characteristic of mucopolysaccharidoses [46,47] (Additional file 11).

### Elastin mRNA and protein are markedly reduced in the SIOD aorta

To test whether the elastin fiber pathology directly arises from altered expression of *ELN* mRNA, we profiled its expression using the Atherosclerosis RT^2^ Profiler^TM^ PCR Array. *ELN* mRNA levels were 121-fold reduced in the aorta of SD120 (p-value = 0.0033; Figure 5A and Additional file 10), and total elastin protein, including soluble and insoluble elastin, was reduced by 63.7% in the aortic tissue lysate of SD120 (Figure 5B).

### SMARCAL1 is expressed in lung myofibroblasts and *ELN* expression is markedly reduced in SIOD lung

Given that impaired elastogenesis may serve as a potential primary and cell autonomous cause of the arteriosclerosis in SIOD, we hypothesized that it might also be a predisposing factor for the emphysematous changes or enlarged air spaces observed in the lungs of SIOD patients. SMARCAL1 was expressed in pneumocytes and lung myofibroblast cells and in the nuclei of cultured normal human lung myofibroblasts (NHLFs) (Figure 4L-P and Additional file 9). Consistent with observations in the aorta, *ELN* mRNA was decreased 156-fold in the lung of SD120 compared to that of unaffected controls (p-value = 0.0023, Figure 5C).

### *ELN* gene mutations are not the cause of the reduced elastogenesis in SIOD

To determine whether the decreased *ELN* mRNA in SD120 arises from mutations in *ELN*, we sequenced the *ELN* gene in the aorta of patient SD120. Among the 34 exons of the *ELN* gene, none were found to have pathogenic mutations. A heterozygous non-synonymous change was found in exon 20 (c.1264G>A, p.Gly422Ser). However, the nucleotide and amino acid of interest are weakly conserved; Align-GVGD and SIFT algorithms predict this variant unlikely to be pathogenic, and this variant has been reported as a single nucleotide polymorphism (SNP, rs2071307) in dbSNP XML build 135 with an average heterozygosity of 0.41. A homozygous intronic change was found in intron 20 (c.1315+17C>T), however this change was not predicted to alter splicing and has been reported as a SNP (rs2856728) with an average heterozygosity of 0.38.

### Expression of *ELN* transcription factors is significantly altered in SIOD aorta and lung

**Figure 6:**
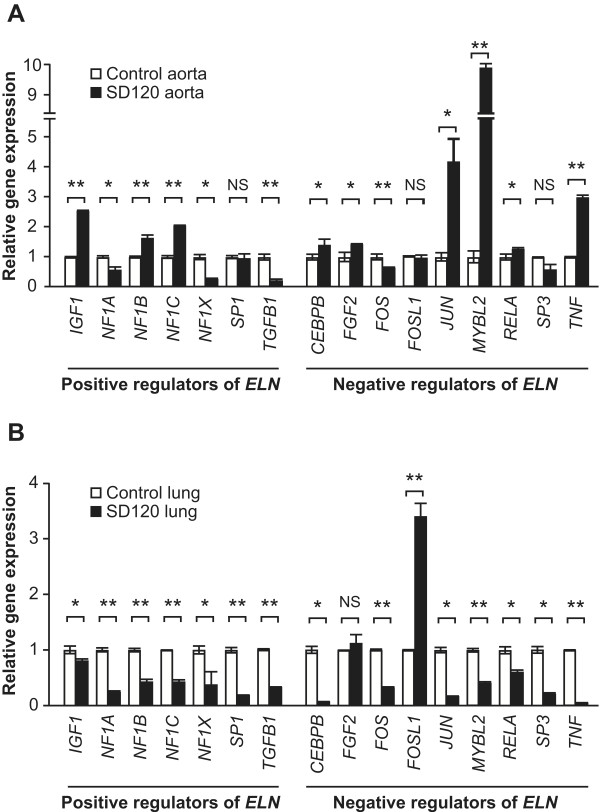
**Expression of known *ELN *transcriptional activators and repressors in patient tissues.** (**A**, **B**) Plots showing the relative mRNA levels of known *ELN* transcriptional activators and repressors in SD120 aorta tissue (A) and in SD120 lung tissue (B) compared to controls. The mRNA levels of three independent replicates were standardized to *GAPDH* mRNA levels and plotted relative to the control. Error bars represent one standard deviation. Abbreviations: NS, not significant; * = p < 0.05, ** = p < 0.01.

We hypothesized that SMARCAL1 deficiency altered the expression of *ELN* either by direct effects on the *ELN* promoter or by alteration of *ELN* transcription factor expression. Compared to controls, the expression of *ELN* repressors *MYBL2*, *JUN*, and *TNF* was increased 9.9-, 4.2- and 3.0-fold, respectively, in the aorta of SD120 (Figure 6A and Additional file 12). Also, in the lung, the expression of all tested *ELN* activators was decreased 1.3- to 5.0-fold and the expression of the negative regulator *FOSL1* was increased 3.4-fold (Figure 6B and Additional file 12).

## Discussion

We have shown that SIOD patients have clinical and histopathological features of impaired vascular and pulmonary elastogenesis. This pathology correlates with decreased *ELN* gene expression and altered expression of *ELN* transcriptional regulators.

Considering the biochemical role of SMARCAL1 [12], we hypothesized that the altered expression of *ELN* arises from direct and indirect effects of SMARCAL1 deficiency on the *ELN* gene. SMARCAL1 deficiency could directly affect *ELN* gene expression by altering the local DNA structure of the *ELN* gene. As an annealing helicase [12], SMARCAL1 might maintain the local DNA structure of transcribed regions and thereby regulate gene expression by modulating transcription factor binding [48]. Alternatively, SMARCAL1 deficiency could indirectly alter *ELN* gene expression by modulating the expression of upstream transcriptional regulators of the *ELN* gene. Consistent with the latter, we observed altered expression of negative and/or positive regulators of *ELN* transcription within the aorta and lung of patient SD120.

The histopathology of the arteries from SIOD patients revealed increased elastic lamellae, increased aortic wall thickness, and fragmented elastin fibers in SIOD. Since SIOD is a multisystem disease characterized by immune deficiency, hypertension, hyperlipidemia, and renal disease [7], we explored cell non-autonomous mechanisms for the basis of the vascular disease. However, as suggested by the lack of recurrence of vascular disease in transplanted kidneys [1,2], we observed onset of vascular ischemia prior to the onset of hypertension and renal failure in some SIOD patients, no detectable inflammatory infiltrate in the aortic wall, and altered distribution of tropoelastin and elastin in a 15-week gestation SIOD fetus. We concluded therefore that a local or cell autonomous mechanism was the most likely cause of the arteriosclerosis.

Such potential mechanisms included osteopontin deficiency, EBP deficiency and impaired elastogenesis. Contrary to the first two hypotheses, the expression of osteopontin mRNA was increased and EBP levels were unaltered in SIOD arteries. Consistent with the third hypothesis, however, we found significantly decreased elastin expression in the aortic tissue of two SIOD patients, which was not a consequence of pathogenic mutations in the *ELN* gene. Based on these findings, we conclude that a primary defect in elastogenesis is a parsimonious mechanism of the vascular and pulmonary disease observed in SIOD.

Elastogenesis is critical for arterial and lung development and maintenance [49,50]. Besides being required for the proper development of the arterial wall, elastin fibers maintain the tensile and elastic integrity of blood vessel walls and regulate the proliferation, migration, and maturation of vascular smooth muscle cells [51,52]. In the lung, elastin fibers are also required for proper development and for elastic recoil [53,54]. Haploinsufficiency for *ELN* causes arterial stenosis and hypertension in supravalvular aortic stenosis and Williams-Beuren syndrome (WBS) as well as mild respiratory symptoms in WBS [55-57]. Elastin deficiency also causes vascular disease, bronchiectasis, and emphysema in cutis laxa, a more severe defect of elastogenesis [24]. Similarly, mice heterozygous for deletion of the *Eln* gene have systemic and pulmonary hypertension, aortic valve disease, and frequent inguinal hernias [30,31]; all of which are observed with increased frequency in SIOD patients [7]. The increase in the number of elastic lamellae and the increased thickness of the tunica media observed in WBS and of mice heterozygous for deletion of the *Eln* gene were also seen in the SIOD arteries [52,58]. Of note, consistent with the later onset of arterial and lung disease in SIOD, the pathology in the SIOD tissue is milder than that typically observed for WBS; nonetheless, these pathological correlations suggest that impaired elastogenesis is a mechanism warranting further investigation as the cause of the arteriosclerosis and emphysematous pulmonary changes of SIOD.

## Conclusions

Vascular and pulmonary disease are common causes of morbidity and mortality in SIOD, and consequently, individuals with SIOD should be evaluated and monitored for the development of vascular and pulmonary disease. Regarding the molecular basis of this pathology, we find that SMARCAL1 deficiency is associated with altered expression of *ELN* transcriptional regulators, severely decreased expression of elastin mRNA and protein and impaired elastogenesis. These observations suggest a mechanism by which SMARCAL1 deficiency affects the pathogenesis of SIOD and await confirmation in additional patients.

## Competing interests

The authors declare that they have no competing interests.

## Authors’ contributions

Experiments and statistical analyses: MM; data interpretation: MM, ZY, PS, CFB; patient ascertainment: ZY, PS, MC, BN, CM, JGW, AKG, DMP, UP, JLA, YA, MB, RB, AB, DB, AB, JC, PC, IC, GD, MSF, PF, SF, HF, EGN, KK, SK, CK, PL, EL, DBL, LM, DRM, DVM, FN, JMS, CNS, LS, NS, AS, DT, DW, JZ, TL, CFB; provision of tissues and technical support for histopathological studies: ZY, PS, GH; manuscript writing: MM, UP, JGW, CFB; study design: CFB. All authors read and approved the final version of the paper.

## Supplementary Material

Additional file 1 **Table S1:** Summary of the patients’ clinical signs and symptoms. Click here for file

Additional file 2 **Table S2:** Lung function parameters for SD16. Click here for file

Additional file 3 **Table S3:** Oligonucleotide primers used in this study. Click here for file

Additional file 4 **Figure S1:** Histopathology of the common iliac and pulmonary arteries of two SIOD patients. Verhoeff van Geison staining of these arteries reveals fragmented and reduced elastin fibers. Arteries are oriented with the tunica adventitia on the left and the tunica intima on the right; the age of death is in parentheses. Scale bars: 50 μm Click here for file

Additional file 5 **Table S4:** Summary of echocardiogram data for SD120. Click here for file

Additional file 6 **Figure S2:** Immunohistochemical detection of smooth muscle actin in the aortic tissue of three SIOD patients. Smooth muscle actin is a marker of smooth muscle cells. Smooth muscle cell hyperplasia was observed in the aortas of SD120 and SD60. Arteries are oriented with the tunica adventitia on the left and the tunica intima on the right; the age of death is in parentheses. Scale bars: 50 μm. Click here for file

Additional file 7 **Figure S3:** Immunohistochemical detection of CD3^+^, CD20^+^, and CD68^+^ cells in aortic tissue of three SIOD patients. CD3, CD20, and CD68 are markers of T cells, B cells, and macrophages, respectively. Inflammatory infiltrates were not observed in the three patients with the exception of macrophages within an atherosclerotic plaque of the aorta of patient SD84. Arteries are oriented with the tunica adventitia on the left and the tunica intima on the right; the age of death is in parentheses. Lymph node tissue sections were used as a positive control. Scale bars: 50 μm. Click here for file

Additional file 8 **Figure S4:***ELN* mRNA expression analysis of the umbilical cord from SIOD and unaffected fetuses at 15-weeks gestation. Plot showing relative *ELN* mRNA expression of the umbilical cord of two age-matched controls compared to that of SD133b by qRT-PCR. The mRNA levels of three independent replicates were standardized to *GAPDH* mRNA levels and plotted relative to the *ELN* mRNA expression of the umbilical cord of SD133b. Error bars represent one standard deviation. ** = p < 0.01. Click here for file

Additional file 9 **Figure S5:** SMARCAL1 is expressed in the vascular smooth muscle (AoSMC), endothelial (HIAEC), and adventitial fibroblast (AoAF) cells of the arterial wall, and in the myofibroblast (NHLF) cells of the lung. (A-H) Photomicrographs showing immunofluorescent localization of SMARCAL1 (red) and α-tubulin (green) in cultured AoSMCs (A), HIAECs (C), AoAFs (E), and NHLFs (G), and photomicrographs showing immunofluorescent localization of SMARCAL1 (red) and the cell-specific markers (green) smooth muscle actin, VE-cadherin, and prolyl 4-hydroxylase in AoSMCs (B), HIAECs (D), AoAFs (F), and NHLFs (H). Scale bars: 10 μm. Click here for file

Additional file 10 **Table S5:** Gene expression changes of atherosclerosis-related genes in SMARCAL1-deficient aorta determined by the Atherosclerosis RT^2^ Profiler^TM^ PCR Array relative to control aorta. Click here for file

Additional file 11 **Figure S6:** Molecular and histopathological analysis of elastin binding protein expression and periodic acid-Schiff staining of SMARCAL1-deficient aorta. (A) Photograph of an immunoblot showing unaltered elastin binding protein (EBP) expression in aortic lysates of SD120 and SD60 compared to a pooled lysate of 49 unaffected individuals; GAPDH was used as a loading control. (B) Periodic acid-Schiff (PAS) staining of the aorta of two patients did not show altered PAS staining compared to age-matched controls. Arteries are oriented with the tunica adventitia on the left and the tunica intima on the right; the age of death is in parentheses. Scale bars: 50 μm. Click here for file

Additional file 12 **Table S6:** Gene expression analysis of transcriptional activators and repressors of *ELN* in SMARCAL1-deficient aorta and lung determined by qRT-PCR relative to control aorta and lung. Click here for file
