# Supplementary material for: Reduced elastogenesis: a clue to the arteriosclerosis and emphysematous changes in Schimke immuno-osseous dysplasia?
Source: Orphanet J Rare Dis. 2012 Sep 22;7:70. doi: 10.1186/1750-1172-7-70 (PMC3568709; doi:10.1186/1750-1172-7-70)
Supplement: Additional file 1 — Table S1: Summary of the patients’ clinical signs and symptoms. [file 1750-1172-7-70-S1.pdf]

Supplementary Table 1. Summary of the patients' clinical signs and symptoms.

Clinical characteristic	Patient	
	SD120	SD16
Sex	Male	Male
Dysmorphism		
Broad low nasal bridge	Yes	No
Bulbous nasal tip	Yes	Yes
Microdontia	Yes	Yes
Hyperpigmented macules	Yes	Yes
Lumbar lordosis	Yes	Yes
Protuberant abdomen	Yes	Yes
Skeleton and growth		
IUGR	Yes	Yes
Disproportionate short stature	Yes	Yes
Spondyloepiphyseal dysplasia	Yes	Yes
Development		
Normal motor development	Mild delay	Yes
Normal language development	Mild delay	Yes
Normal social development	Yes	Yes
Normal cognitive development	Yes	Yes
Normal school performance	Yes	Yes
Hematology		
Lymphopenia	Yes	Yes
Neutropenia	No	Yes
Anemia	No	No
Thrombocytopenia	No	No
Immunology		
Recurrent infections	No	No
Circulating T cell deficiency	Yes	Unknown
Circulating B cell deficiency	No	Unknown
Nephrology		
FSGS	Yes	Yes
Progressive renal failure	Yes	Yes
Renal transplant	No	Yes
Hypertension	Yes	Yes
Cardiovascular		
Left ventricular hypertrophy	Yes	No
Aortic valve disease	No	Yes
TIAs	Yes	No

Arteriosclerosis	Yes	No
Cerebral infarcts	No	No
Cerebral moyamoya	No	No
Migraines	Yes	No
Pulmonary		
Panlobular emphysema	No	Yes
Dyspnea	No	Yes
Restrictive lung disease	Yes	Yes

Abbreviations: FSGS, focal segmental glomerulosclerosis; IUGR, intrauterine growth retardation; TIA, transient ischemic attack.
