# Supplementary material for: Reduced elastogenesis: a clue to the arteriosclerosis and emphysematous changes in Schimke immuno-osseous dysplasia?
Source: Orphanet J Rare Dis. 2012 Sep 22;7:70. doi: 10.1186/1750-1172-7-70 (PMC3568709; doi:10.1186/1750-1172-7-70)
Supplement: Additional file 2 — Table S2: Lung function parameters for SD16. [file 1750-1172-7-70-S2.pdf]

Supplementary Table 2. Lung function parameters for SD16

Parameter	Unit	SD16 Measure	Reference Measure[A]	% Reference
Spirometry				
FVC	L	1.72	2.82	61
FEV_1	L	1.37	2.63	52
FEV_1/FVC	%	79	87	91
$FEF_{25\text{-}75\%}$	L/sec	1.48	3.60	41
$FEF_{50\%}$	L/sec	4.37	2.55	171
$FEF_{75\%}$	L/sec	0.41	0.96	43
PEF	L/sec	6.48	5.67	114
FIVC	L	1.72		
PIF	L/sec	4.64		
Lung Volumes				
VC	L	1.77	2.82	63
TLC	L	2.58	3.21	80
RV	L	0.81	0.85	96
RV/TLC	%	31	29	110
FRC	L	1.15	1.49	77
IC	L	1.43		
ERV	L	0.36		
Diffusion Capacity				
DL_{CO}	mL/mmHg/min	4.30	27.50	15
DL_{CO} adj for [Hb]	mL/mmHg/min	4.80	27.50	17
V_A	L	2.38	3.21	74
DL_{CO} adj for V_A	mL/mmHg/min	5.40	27.50	20

[A]Reference values generated from normal subjects at the University of Alberta and follows the American Thoracic Society (ATS) criteria, that is, matched to the patient's age, gender, height, weight, body mass index and ethnicity [39].

Abbreviations: adj, adjusted; DL_{CO}, carbon monoxide diffusing capacity; ERV; expiratory reserve volume; FEF, forced expiratory flow; $FEF_{25\text{-}75\%}$, average forced expiratory flow from 25% to 75% of vital capacity; $FEF_{50\%}$, forced expiratory flow at 50% of vital capacity; $FEF_{75\%}$, forced expiratory flow at 75% of vital capacity; FEV_1, forced expiratory volume in one second; FIVC, forced inspiratory vital capacity; FVC, forced vital capacity; FRC, functional residual capacity; [Hb], hemoglobin concentration; IC, inspiratory capacity; IVC, inspiratory vital capacity; L, liters; L/sec, liters per second; mL, milliliters; mmHg, millimeters of mercury; min, minute; PEF, peak expiratory flow;

PIF, peak inspiratory flow; %, percent; RV; residual volume; TLC, total lung capacity; V_A, alveolar volume; VC, vital capacity.
