# Supplementary material for: Reduced elastogenesis: a clue to the arteriosclerosis and emphysematous changes in Schimke immuno-osseous dysplasia?
Source: Orphanet J Rare Dis. 2012 Sep 22;7:70. doi: 10.1186/1750-1172-7-70 (PMC3568709; doi:10.1186/1750-1172-7-70)
Supplement: Additional file 3 — Table S3: Oligonucleotide primers used in this study. [file 1750-1172-7-70-S3.pdf]

Supplementary Table 2. Sequences of oligonucleotide primers used in this study.

Primer	Sequence
SMARCAL1 expression	
hGAPDH-cDNA-F	CTTTTGCGTCGCCAGCCGAG
hGAPDH-cDNA-R	GGTGACCAGGCGCCCAATACG
SMARCAL1-cDNA1-F	CCTCTACAAGGACCCAAAGCAGCAG
SMARCAL1-cDNA1-R	TCCAGGGTGTCTCCCATGTTCTGG
Cell-specific markers	
ACTA2-F	CGGGAATCCTGTGAAGCAGCTCC
ACTA2-R	ATCACCCCCTGATGTCTGGGACG
CDH5-F	TCGTCGGCTGTGGGGACCTC
CDH5-R	GTCGCCCCGCAAGATGCTGT
P4HA3-F	CCCTGGGTTCCCAGCCCACT
P4HA3-R	GAGGGCAGCAATGCGGTGGT
ELN expression	
ELN-F	GGGCTCTCGGTGGAGTAGGCA
ELN-R	CAGCAGCACCGTATTTAGCTGCTTT
ELN-91 bp-F	AAATACGGTGCTGCTGGCCTT
ELN-91 bp-R	ACAATCCGAAGCCAGGTCTTG
Positive regulators of ELN	
IGF1-F	TGTGACATTGCTCTCAACATCTCCC
IGF1-R	GACATGGTGTGCATCTTCACCTTCA
NF1A-F	AGGGCTCTGTGTCCAACCCCA
NF1A-R	GCCCAAATGTCCATTTTCTGGCTGG
NF1B-F	TCCAAGCCACAATGATCCTGCC
NF1B-R	AGTTCCCTGGGTTATGGGCGTTCT
NF1C-F	CTTCGTGCGTGAGCGAGATGC
NF1C-R	ACACGCCGGAGGTGACAAAGC
NF1X-F	CGTCCAGCCACATCACATTGGAGT
NF1X-R	AACTTAAGTGCCCGTTGGGCAG
SP1-F	CTCGTCAGCGTCCGCGTTTTTC
SP1-R	GGAGTGGACTCATCCTTACCGCTC
TGFB1-F	CTGCAAGTGGACATCAACGGGTT
TGFB1-R	GCACGCAGCAGTTCTTCTCCGT
Negative regulators of ELN	
CEBPB-F	TTCATGCAACGCTGGTGGCCTG
CEBPB-R	TCCGCCTCGTAGTAGAAGTTGGCCA
FGF2-F	GACCCCAAGCGGCTGTACTGC
FGF2-R	TTGTAGCTTGATGTGAGGGTCGC
FOS-F	GGCTTTGCCTAACCGCCACG
FOS-R	GTGCAGAAGTCCTGCGCGTTGA
FOSL1-F	CGCCTCCAGGGGTACGTCGAA
FOSL1-R	TTCCAGTTTGTCAGTCTCCGCCTGC
JUN-F	AGGGTCCGCACTGATCCGCT
JUN-R	CTCGGAGTCCGCAGGCGAAC

MYBL2-F	TGCGTTCTGAGGCTGGCATCG
MYBL2-R	TGAAGGGGCAGTTGTCGGCAAG
RELA-F	AGGCTCCTGTGCGTGTCTCCAT
RELA-R	GTCGGTGGGTCCGCTGAAAGGACT
SP3-F	ACCTACTTTCCTTGGCAGGAAGCT
SP3-R	TCACCAGAGTTGGGAAGAAGGCA
TNF-F	GAGGCCAAGCCCTGGTATG
TNF-R	CGGGCCGATTGATCTCAGC
