# Supplementary figures and images for: Reduced elastogenesis: a clue to the arteriosclerosis and emphysematous changes in Schimke immuno-osseous dysplasia?
Source: Orphanet J Rare Dis. 2012 Sep 22;7:70. doi: 10.1186/1750-1172-7-70 (PMC3568709; doi:10.1186/1750-1172-7-70)

*Common Iliac Artery*

Control (0.08 yr)

SD120 (5.4 yr)

SD60 (13.7 yr)

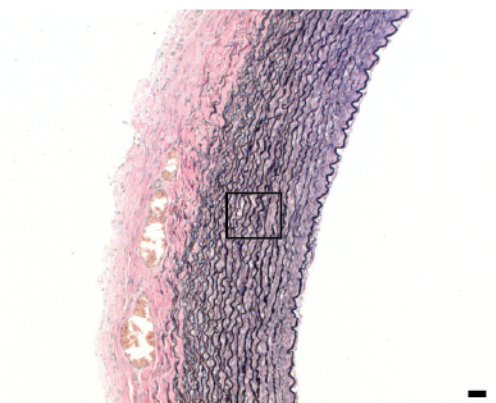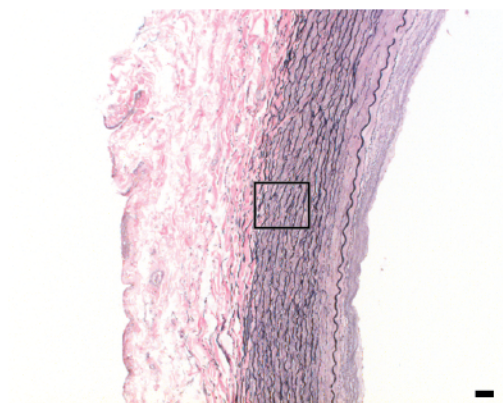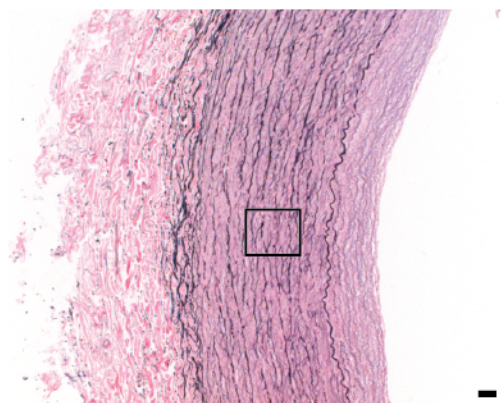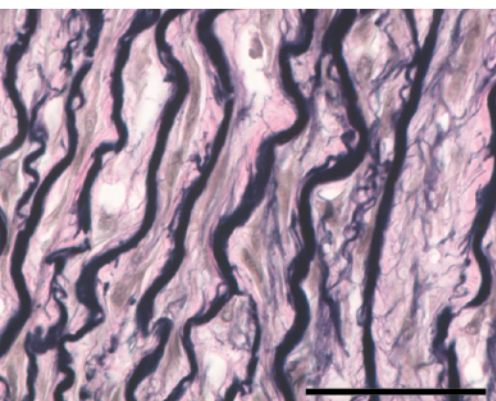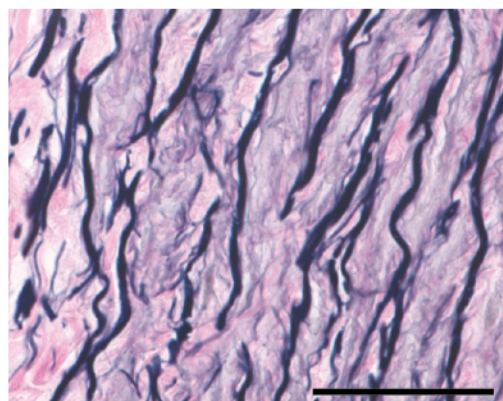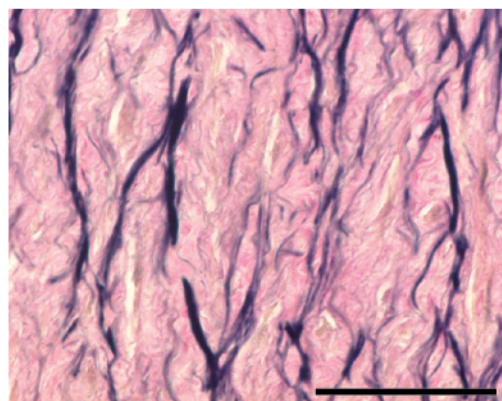

*Pulmonary Artery*

Control (0.08 yr)

SD120 (5.4 yr)

SD60 (13.7 yr)

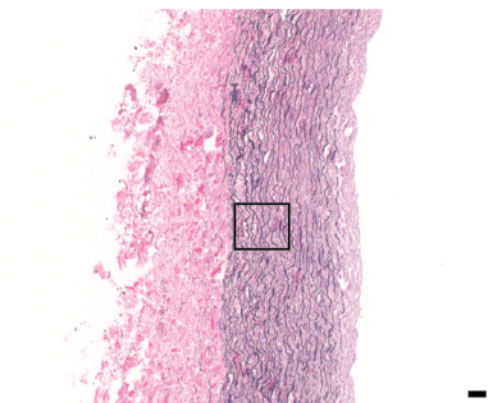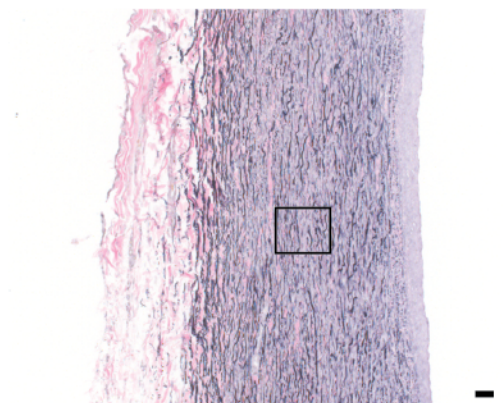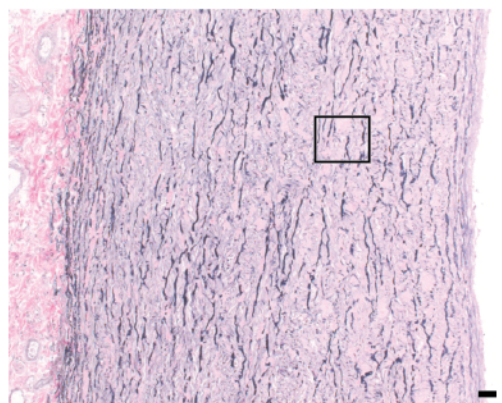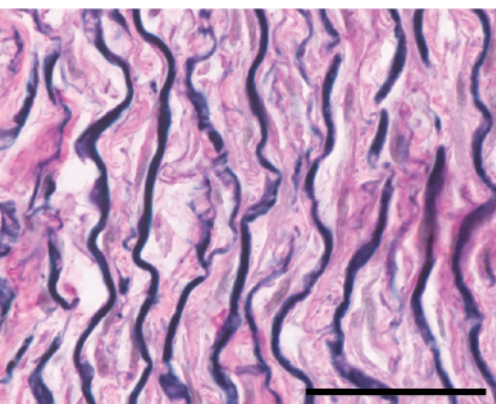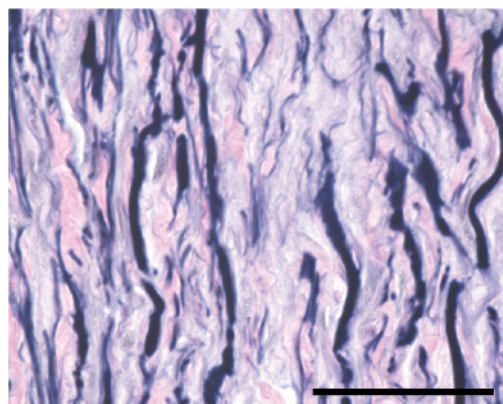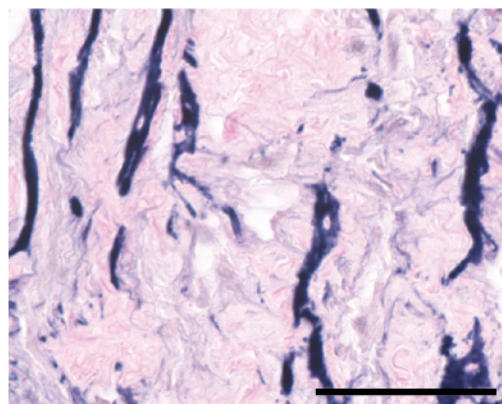

Supplement: Additional file 4 — Figure S1: Histopathology of the common iliac and pulmonary arteries of two SIOD patients. Verhoeff van Geison staining of these arteries reveals fragmented and reduced elastin fibers. Arteries are oriented with the tunica adventitia on the left and the tunica intima on the right; the age of death is in parentheses. Scale bars: 50 μm [file 1750-1172-7-70-S4.pdf]

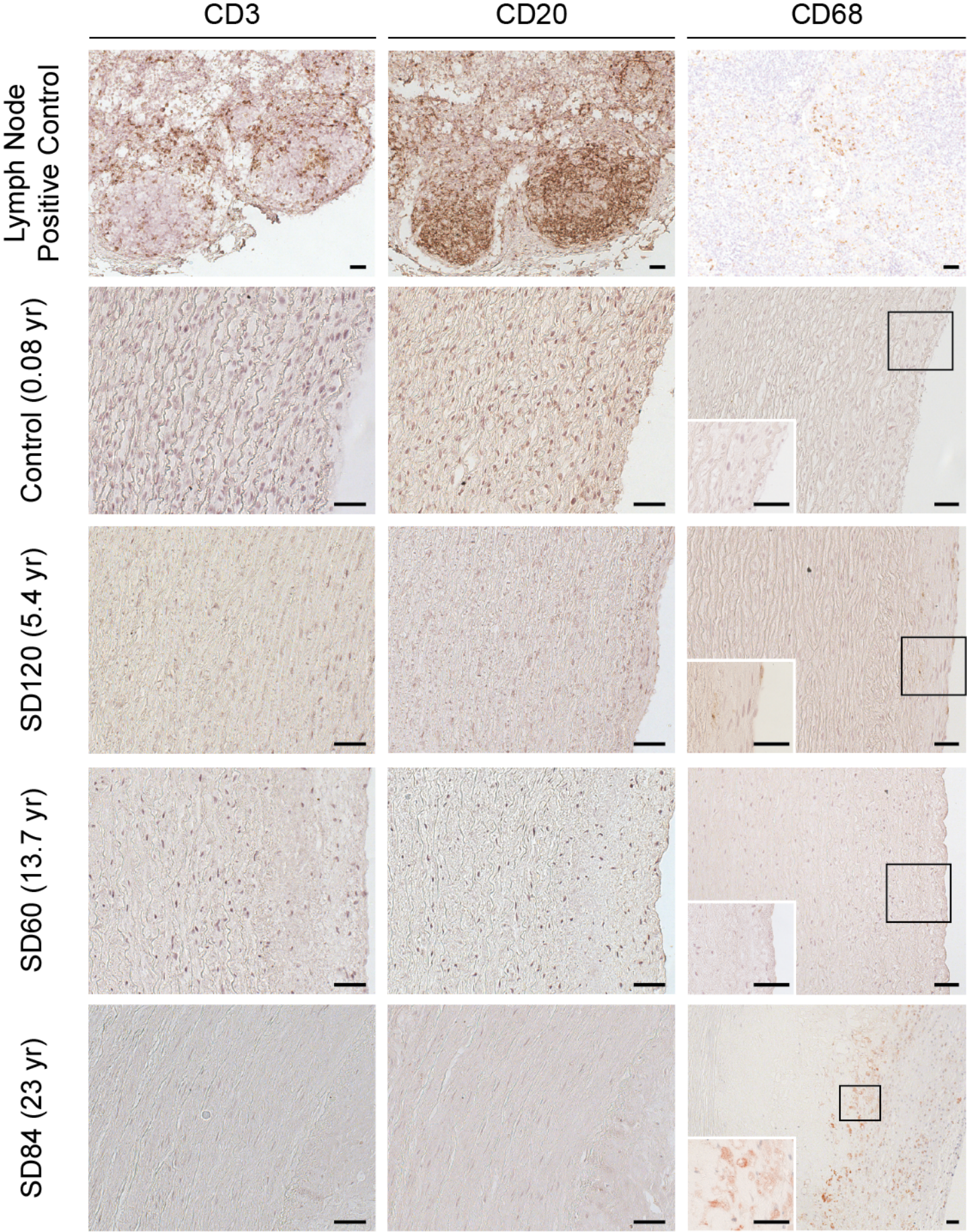

Supplement: Additional file 7 — Figure S3: Immunohistochemical detection of CD3+, CD20+, and CD68+ cells in aortic tissue of three SIOD patients. CD3, CD20, and CD68 are markers of T cells, B cells, and macrophages, respectively. Inflammatory infiltrates were not observed in the three patients with the exception of macrophages within an atherosclerotic plaque of the aorta of patient SD84. Arteries are oriented with the tunica adventitia on the left and the tunica intima on the right; the age of death is in parentheses. Lymph node tissue sections were used as a positive control. Scale bars: 50 μm. [file 1750-1172-7-70-S7.pdf]

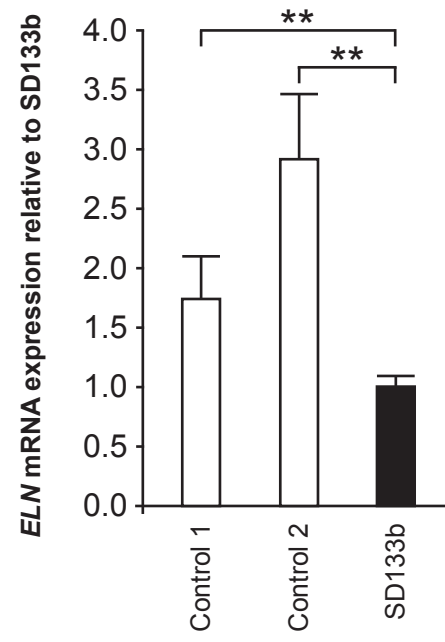

Supplement: Additional file 8 — Figure S4:ELN mRNA expression analysis of the umbilical cord from SIOD and unaffected fetuses at 15-weeks gestation. Plot showing relative ELN mRNA expression of the umbilical cord of two age-matched controls compared to that of SD133b by qRT-PCR. The mRNA levels of three independent replicates were standardized to GAPDH mRNA levels and plotted relative to the ELN mRNA expression of the umbilical cord of SD133b. Error bars represent one standard deviation. ** = p < 0.01. [file 1750-1172-7-70-S8.pdf]

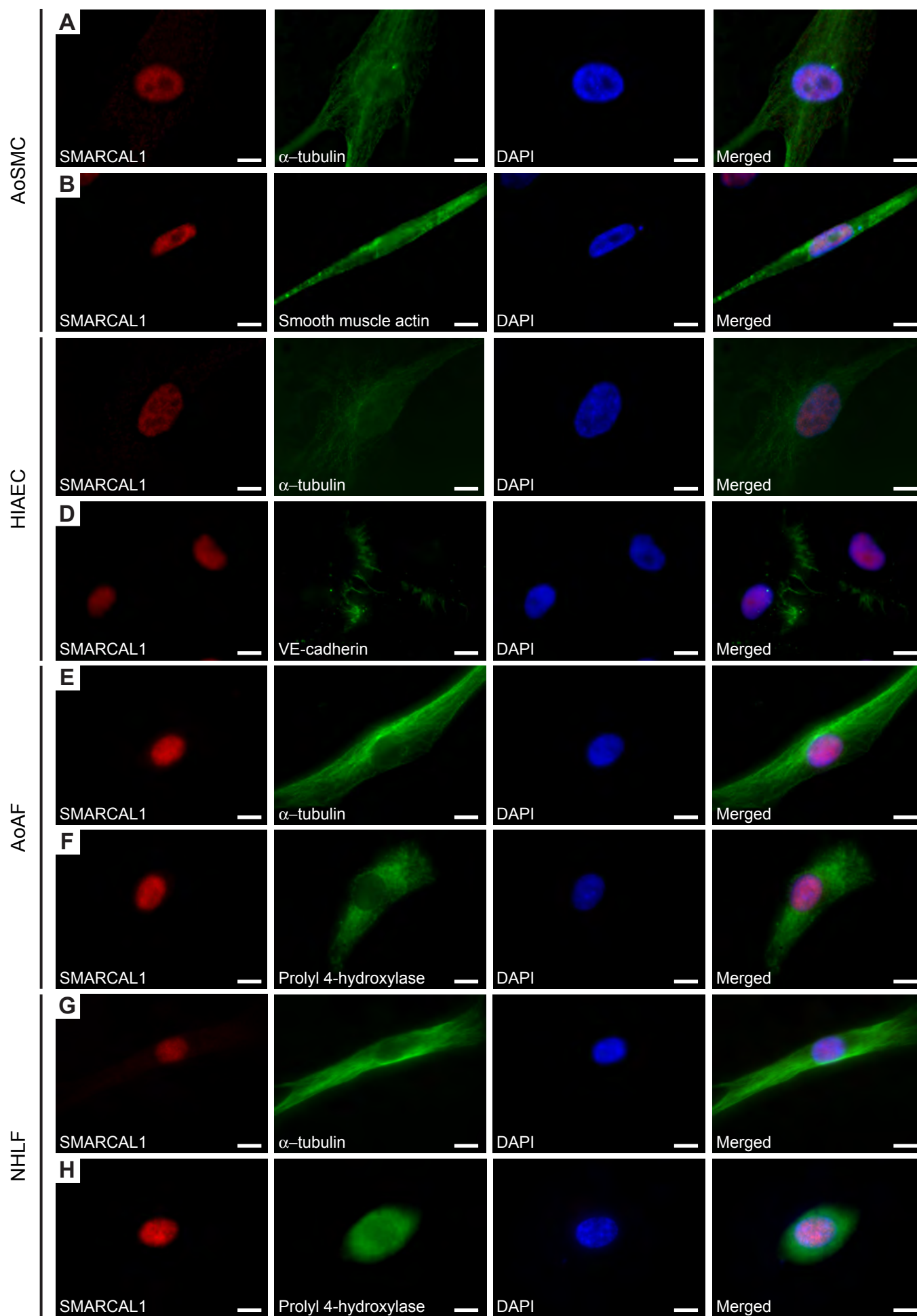

Supplement: Additional file 9 — Figure S5: SMARCAL1 is expressed in the vascular smooth muscle (AoSMC), endothelial (HIAEC), and adventitial fibroblast (AoAF) cells of the arterial wall, and in the myofibroblast (NHLF) cells of the lung. (A-H) Photomicrographs showing immunofluorescent localization of SMARCAL1 (red) and α-tubulin (green) in cultured AoSMCs (A), HIAECs (C), AoAFs (E), and NHLFs (G), and photomicrographs showing immunofluorescent localization of SMARCAL1 (red) and the cell-specific markers (green) smooth muscle actin, VE-cadherin, and prolyl 4-hydroxylase in AoSMCs (B), HIAECs (D), AoAFs (F), and NHLFs (H). Scale bars: 10 μm. [file 1750-1172-7-70-S9.pdf]

**A**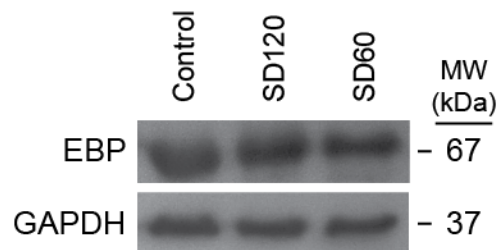**B**

Periodic Acid-Schiff

Control (0.08 yr)

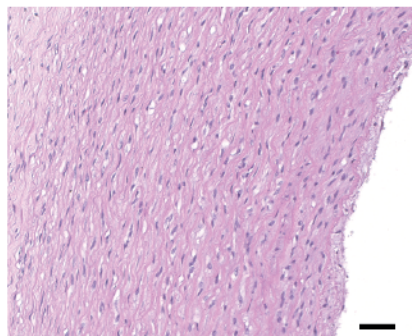

Control (5.0 yr)

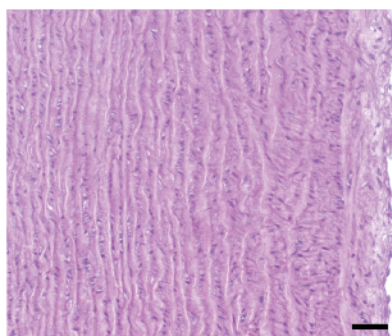

Control (15.0 yr)

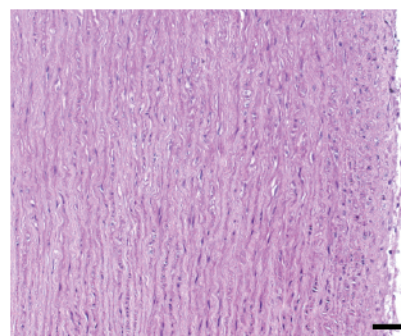

SD120 (5.4 yr)

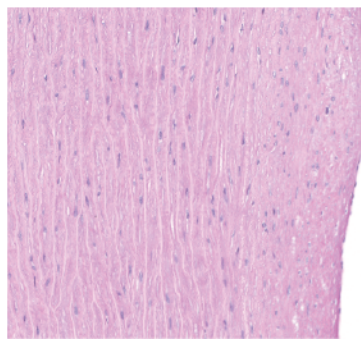

SD60 (13.7 yr)

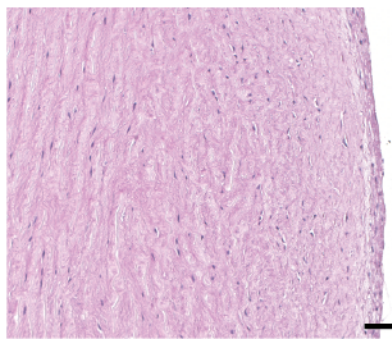

SD84 (23 yr)

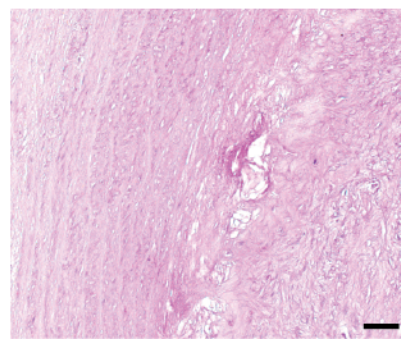

Supplement: Additional file 11 — Figure S6: Molecular and histopathological analysis of elastin binding protein expression and periodic acid-Schiff staining of SMARCAL1-deficient aorta. (A) Photograph of an immunoblot showing unaltered elastin binding protein (EBP) expression in aortic lysates of SD120 and SD60 compared to a pooled lysate of 49 unaffected individuals; GAPDH was used as a loading control. (B) Periodic acid-Schiff (PAS) staining of the aorta of two patients did not show altered PAS staining compared to age-matched controls. Arteries are oriented with the tunica adventitia on the left and the tunica intima on the right; the age of death is in parentheses. Scale bars: 50 μm. [file 1750-1172-7-70-S11.pdf]
