# Supplementary material for: Reduced elastogenesis: a clue to the arteriosclerosis and emphysematous changes in Schimke immuno-osseous dysplasia?
Source: Orphanet J Rare Dis. 2012 Sep 22;7:70. doi: 10.1186/1750-1172-7-70 (PMC3568709; doi:10.1186/1750-1172-7-70)
Supplement: Additional file 5 — Table S4: Summary of echocardiogram data for SD120. [file 1750-1172-7-70-S5.pdf]

Supplementary Table 4. Summary of echocardiogram data for SD120

Level of the aortic root[A]	Diameter (mm)	Z-score[B]
Aortic valve	11.1	0.27
Sinus of Valsalva	14.8	0.41
Sinotubular junction	14.7	2.09
Ascending aorta	15.3	1.97

[A]Measurements of the aorta were obtained in accordance to the American Society of Echocardiogram guidelines in real time and confirmed post mortem [36].

[B]Body surface area and Z scores were calculated using the Haycock and Halifax formulae, respectively [37, 38]. The normal range is considered to be within -2 to 2 standard deviations from the mean diameter.
