# Supplementary material for: Reduced elastogenesis: a clue to the arteriosclerosis and emphysematous changes in Schimke immuno-osseous dysplasia?
Source: Orphanet J Rare Dis. 2012 Sep 22;7:70. doi: 10.1186/1750-1172-7-70 (PMC3568709; doi:10.1186/1750-1172-7-70)
Supplement: Additional file 6 — Figure S2: Immunohistochemical detection of smooth muscle actin in the aortic tissue of three SIOD patients. Smooth muscle actin is a marker of smooth muscle cells. Smooth muscle cell hyperplasia was observed in the aortas of SD120 and SD60. Arteries are oriented with the tunica adventitia on the left and the tunica intima on the right; the age of death is in parentheses. Scale bars: 50 μm. [file 1750-1172-7-70-S6.pdf]

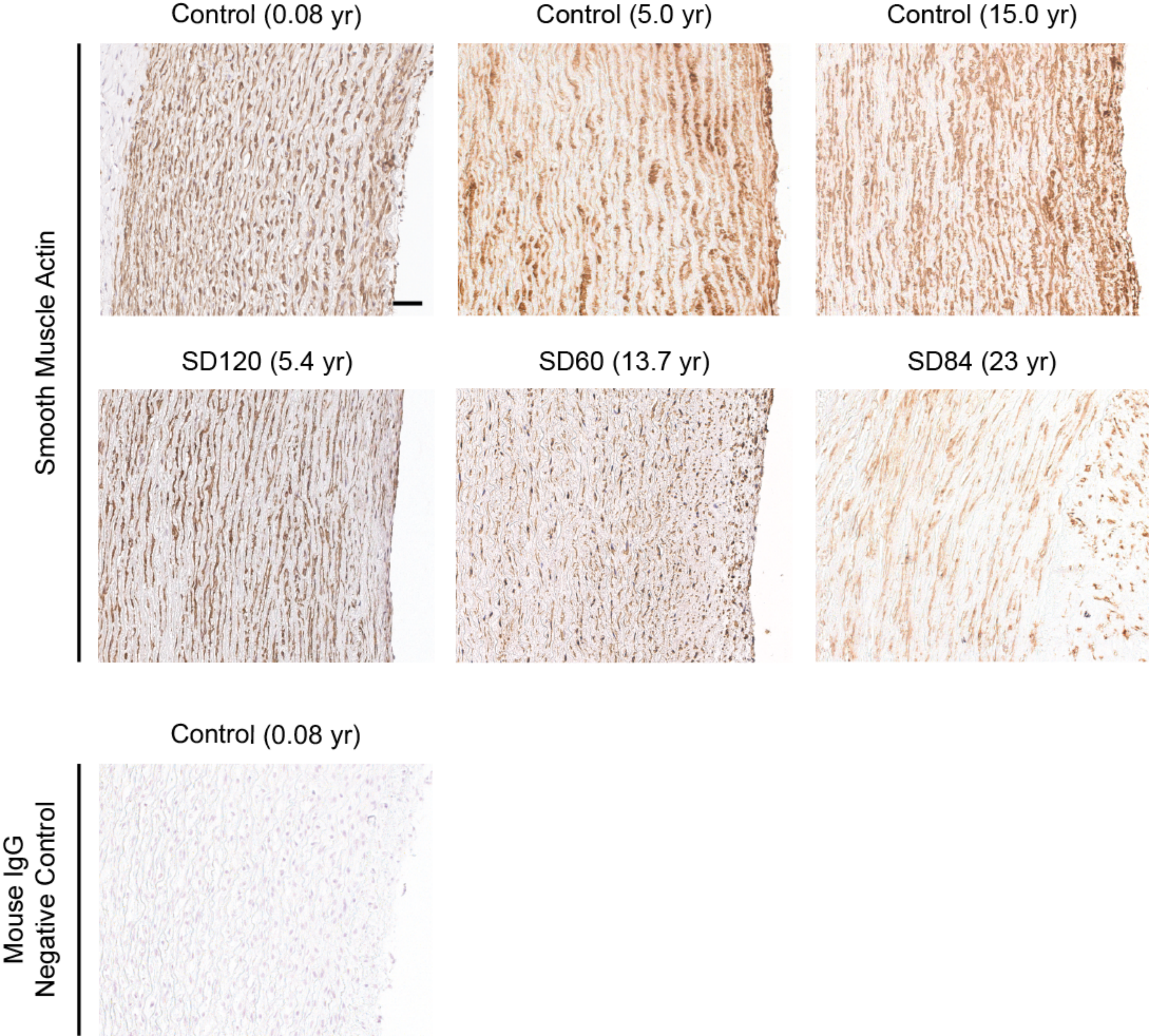

Control (0.08 yr)
Control (5.0 yr)
Control (15.0 yr)
Smooth Muscle Actin
SD120 (5.4 yr)
SD60 (13.7 yr)
SD84 (23 yr)
Control (0.08 yr)
Mouse IgG
Negative Control
