# Supplementary material for: Reduced elastogenesis: a clue to the arteriosclerosis and emphysematous changes in Schimke immuno-osseous dysplasia?
Source: Orphanet J Rare Dis. 2012 Sep 22;7:70. doi: 10.1186/1750-1172-7-70 (PMC3568709; doi:10.1186/1750-1172-7-70)
Supplement: Additional file 10 — Table S5: Gene expression changes of atherosclerosis-related genes in SMARCAL1-deficient aorta determined by the Atherosclerosis RT2 ProfilerTM PCR Array relative to control aorta. [file 1750-1172-7-70-S10.pdf]

Supplementary Table 5. Gene expression changes of atherosclerosis-related genes in SMARCAL1-deficient aorta determined by the Atherosclerosis RT^2 Profiler™ PCR Array relative to control aorta.

Gene	Normalized Fold Change	P-value
ABCA1	25.9	8.9×10^{-4}
ACE	2.7	1.1×10^{-3}
ADFP	-1.1	4.0×10^{-1}
APOA1	1.5	1.1×10^{-1}
APOB	2.2	1.1×10^{-1}
APOE	7.5	1.1×10^{-5}
BAX	3.4	4.3×10^{-4}
BCL2	2.5	6.7×10^{-4}
BCL2A1	3.6	2.0×10^{-2}
BCL2L1	-1.3	2.6×10^{-2}
BID	1.3	1.8×10^{-1}
BIRC3	3.3	1.7×10^{-3}
CCL2	1.6	2.1×10^{-3}
CCL5	-4.4	2.5×10^{-4}
CCR1	6.7	1.9×10^{-5}
CCR2	-2.8	7.3×10^{-2}
CD44	1.2	6.1×10^{-2}
CDH5	-1.3	4.1×10^{-2}
CFLAR	3.2	9.3×10^{-5}
COL3A1	1.4	1.7×10^{-3}
CSF1	8.4	1.2×10^{-4}
CSF2	2.5	3.9×10^{-1}
CTGF	-2.6	2.7×10^{-5}
EGR1	-7.8	1.8×10^{-4}
ELN	-121.5	3.3×10^{-3}
ENG	-1.9	6.4×10^{-5}
FABP3	1.3	4.7×10^{-2}
FAS	4.8	3.0×10^{-3}
FGA	96.4	1.2×10^{-2}
FGF2	2.6	3.0×10^{-5}
FN1	1.4	9.8×10^{-3}
HBEGF	3.8	4.1×10^{-3}
ICAM1	1.9	6.1×10^{-5}
IFNAR2	2.1	2.6×10^{-2}
IFNG	-5.1	2.9×10^{-2}
IL1A	2.3	2.7×10^{-1}
IL1R1	8.1	8.6×10^{-4}
IL1R2	-2.2	1.0×10^{-1}
IL2	4.0	1.1×10^{-1}

IL3	2.3	5.6×10^{-1}
IL4	4.3	2.5×10^{-1}
IL5	8.4	9.5×10^{-4}
ITGA2	-1.3	7.0×10^{-1}
ITGA5	-4.0	1.2×10^{-3}
ITGAX	1.6	4.4×10^{-2}
ITGB2	2.5	1.5×10^{-3}
KDR	2.7	1.3×10^{-2}
KLF2	-1.2	1.5×10^{-1}
LAMA1	2.4	2.5×10^{-2}
LDLR	1.1	5.6×10^{-1}
LIF	-1.9	1.2×10^{-2}
LPA	5.7	2.6×10^{-1}
LPL	1.9	5.7×10^{-3}
MMP1	-4.1	1.8×10^{-2}
MMP3	-30.1	2.6×10^{-4}
MSR1	22.3	1.3×10^{-1}
NFKB1	-1.4	7.0×10^{-2}
NOS3	-1.7	2.5×10^{-3}
NPY	-3.8	2.5×10^{-3}
NR1H3	3.1	1.1×10^{-2}
PDGFA	-4.3	8.8×10^{-5}
PDGFB	-1.2	2.9×10^{-1}
PDGFRB	1.7	7.0×10^{-1}
PPARA	3.8	1.2×10^{-3}
PPARD	2.6	2.8×10^{-2}
PPARG	3.4	1.5×10^{-3}
PTGS1	1.0	8.8×10^{-1}
RXRA	-1.8	8.7×10^{-3}
SELE	5.3	8.5×10^{-1}
SELL	1.1	2.9×10^{-1}
SELPLG	2.4	9.8×10^{-3}
SERPINB2	-1.8	6.8×10^{-2}
SERPINE1	-14.2	4.8×10^{-5}
SOD1	1.3	1.1×10^{-2}
SPP1	2.6	1.5×10^{-3}
TGFB1	-1.3	1.3×10^{-2}
TGFB2	-1.6	1.4×10^{-2}
THBS4	-2.5	2.6×10^{-3}
TNC	-2.1	1.8×10^{-3}
TNF	-1.6	3.0×10^{-2}
TNFAIP3	3.4	4.6×10^{-3}
VCAM1	2.0	6.9×10^{-5}
VEGFA	-2.5	1.6×10^{-4}
VWF	1.1	4.6×10^{-1}
