# Supplementary material for: Reduced elastogenesis: a clue to the arteriosclerosis and emphysematous changes in Schimke immuno-osseous dysplasia?
Source: Orphanet J Rare Dis. 2012 Sep 22;7:70. doi: 10.1186/1750-1172-7-70 (PMC3568709; doi:10.1186/1750-1172-7-70)
Supplement: Additional file 12 — Table S6: Gene expression analysis of transcriptional activators and repressors of ELN in SMARCAL1-deficient aorta and lung determined by qRT-PCR relative to control aorta and lung. [file 1750-1172-7-70-S12.pdf]

Supplementary Table 6. Gene expression analysis of transcriptional activators and repressors of *ELN* in SMARCAL1-deficient aorta and lung determined by qRT-PCR relative to control aorta and lung, respectively.

Tissue and Gene	Normalized Fold Change	P-value	Reference
**Aorta**			
Positive Regulators			
IGF1	2.5	1.0×10^{-4}	[1-3]
NF1A	-1.7	4.3×10^{-2}	[4]
NF1B	1.7	3.0×10^{-3}	[4]
NF1C	2.1	1.1×10^{-4}	[4]
NF1X	-3.3	5.0×10^{-2}	[4]
SP1	1.0	7.3×10^{-1}	[2, 5]
TGFB1	-5.0	1.9×10^{-3}	[6]
Negative Regulators			
CEBPB	1.4	4.6×10^{-2}	[7]
FGF2	1.4	4.1×10^{-2}	[8-11]
FOS	-1.4	2.0×10^{-2}	[10]
FOSL1	1.0	8.6×10^{-1}	[9-11]
JUN	4.2	1.5×10^{-2}	[9]
MYBL2	9.9	1.0×10^{-5}	[12]
RELA	1.3	3.2×10^{-2}	[7]
SP3	-1.7	1.4×10^{-1}	[2]
TNF	3.0	6.8×10^{-3}	[13]
**Lung**			
Positive Regulators			
IGF1	-1.3	2.8×10^{-2}	[1-3]
NF1A	-5.0	3.0×10^{-5}	[4]
NF1B	-2.5	5.7×10^{-4}	[4]
NF1C	-2.5	6.1×10^{-4}	[4]
NF1X	-2.5	3.4×10^{-2}	[4]
SP1	-5.0	1.1×10^{-3}	[2, 5]
TGFB1	-3.3	1.8×10^{-3}	[6]
Negative Regulators			
CEBPB	-10.0	3.0×10^{-2}	[7]
FGF2	1.1	2.9×10^{-1}	[8-11]
FOS	-3.3	2.0×10^{-5}	[10]
FOSL1	3.4	3.2×10^{-3}	[9-11]
JUN	-5.0	2.4×10^{-2}	[9]
MYBL2	-2.5	8.2×10^{-3}	[12]
RELA	-1.7	3.7×10^{-2}	[7]
SP3	-5.0	2.0×10^{-2}	[2]
TNF	-20.0	2.6×10^{-3}	[13]

REFERENCES

1. Rich CB, Ewton DZ, Martin BM, Florini JR, Bashir M, Rosenbloom J, Foster JA: **IGF-I regulation of elastogenesis: comparison of aortic and lung cells.** *Am J Physiol* 1992, **263:**L276-282.
2. Conn KJ, Rich CB, Jensen DE, Fontanilla MR, Bashir MM, Rosenbloom J, Foster JA: **Insulin-like growth factor-I regulates transcription of the elastin gene through a putative retinoblastoma control element. A role for Sp3 acting as a repressor of elastin gene transcription.** *J Biol Chem* 1996, **271:**28853-28860.
3. Wolfe BL, Rich CB, Goud HD, Terpstra AJ, Bashir M, Rosenbloom J, Sonenshein GE, Foster JA: **Insulin-like growth factor-I regulates transcription of the elastin gene.** *J Biol Chem* 1993, **268:**12418-12426.
4. Degterev A, Foster JA: **The role of NF-1 factors in regulation of elastin gene transcription.** *Matrix Biol* 1999, **18:**295-307.
5. Jensen DE, Rich CB, Terpstra AJ, Farmer SR, Foster JA: **Transcriptional regulation of the elastin gene by insulin-like growth factor-I involves disruption of Sp1 binding. Evidence for the role of Rb in mediating Sp1 binding in aortic smooth muscle cells.** *J Biol Chem* 1995, **270:**6555-6563.
6. Kuang PP, Zhang XH, Rich CB, Foster JA, Subramanian M, Goldstein RH: **Activation of elastin transcription by transforming growth factor-beta in human lung fibroblasts.** *Am J Physiol Lung Cell Mol Physiol* 2007, **292:**L944-952.

7. Kuang PP, Goldstein RH: **Regulation of elastin gene transcription by interleukin-1 beta-induced C/EBP beta isoforms.** *Am J Physiol Cell Physiol* 2003, **285:**C1349-1355.

8. Rich CB, Nugent MA, Stone P, Foster JA: **Elastase release of basic fibroblast growth factor in pulmonary fibroblast cultures results in down-regulation of elastin gene transcription. A role for basic fibroblast growth factor in regulating lung repair.** *J Biol Chem* 1996, **271:**23043-23048.

9. Rich CB, Fontanilla MR, Nugent M, Foster JA: **Basic fibroblast growth factor decreases elastin gene transcription through an AP1/cAMP-response element hybrid site in the distal promoter.** *J Biol Chem* 1999, **274:**33433-33439.

10. Carreras I, Rich CB, Jaworski JA, Dicamillo SJ, Panchenko MP, Goldstein R, Foster JA: **Functional components of basic fibroblast growth factor signaling that inhibit lung elastin gene expression.** *Am J Physiol Lung Cell Mol Physiol* 2001, **281:**L766-775.

11. Carreras I, Rich CB, Panchenko MP, Foster JA: **Basic fibroblast growth factor decreases elastin gene transcription in aortic smooth muscle cells.** *J Cell Biochem* 2002, **85:**592-600.

12. Hofmann CS, Wang X, Sullivan CP, Toselli P, Stone PJ, McLean SE, Mecham RP, Schreiber BM, Sonenshein GE: **B-Myb represses elastin gene expression in aortic smooth muscle cells.** *J Biol Chem* 2005, **280:**7694-7701.

13. Kahari VM, Chen YQ, Bashir MM, Rosenbloom J, Uitto J: **Tumor necrosis factor-alpha down-regulates human elastin gene expression. Evidence for the**

role of AP-1 in the suppression of promoter activity. *J Biol Chem* 1992, **267:**26134-26141.
